# Constructing Dissolution–Resistant Interphases for Long‐Life Sodium‐Ion Batteries at Elevated Temperatures

**DOI:** 10.1002/advs.202502860

**Published:** 2025-05-08

**Authors:** Wenting Deng, Xiaofan Du, Gaojie Xu, Shitao Wang, Li Du, Tiantian Dong, Rongxian Wu, Chuanchuan Li, Zhaolin Lv, Jiangwei Ju, Xinhong Zhou, Guanglei Cui

**Affiliations:** ^1^ College of Chemistry and Molecular Engineering Qingdao University of Science and Technology Qingdao 266042 China; ^2^ Qingdao Industrial Energy Storage Research Institute Qingdao Institute of Bioenergy and Bioprocess Technology Chinese Academy of Science Qingdao 266101 China; ^3^ Shandong Energy Institute Qingdao 266101 China; ^4^ Qingdao New Energy Shandong Laboratory Qingdao 266101 China

**Keywords:** carbonate electrolyte, dissolution–resistant interphase, elevated temperature, functional additive, sodium‐ion batteries

## Abstract

Rechargeable sodium‐ion batteries (SIBs) utilizing NaPF_6_‐carbonate electrolytes consistently exhibit unsatisfactory cycle life at elevated temperatures, posing a significant challenge for their large‐scale commercialization. This is mainly caused by the instability of interphase layers at elevated temperatures, especially the high solubility of interphase components (especially NaF) in carbonate solvents. In this study, a novel additive of sodium difluorobis(oxalato) phosphate (NaDFBOP) is synthesized and introduced into NaPF_6_‐carbonate electrolytes to enhance the cycle life of commercial SIBs composed of NaNi_1/3_Fe_1/3_Mn_1/3_O_2_ (NFM) cathode and hard carbon (HC) anode, particularly at 50 °C. Specifically, the NaDFBOP enables NFM/HC SIBs to retain 85.45% of initial capacity after 1000 cycles at 30 °C and 90.76% after 500 cycles at 50 °C. Theoretical calculations reveal that DFBOP⁻ anions enter the first solvation shell of Na^+^, and NaDFBOP exhibits a strong propensity for decomposition. Characterizations suggest that NaDFBOP favors the formation of dissolution–resistant robust interphase layers enriched of dissolution‐resistant oxalate‐containing species and inorganic NaF, which have strong mutual binding energy. This work underscores the critical importance of designing functional additives and constructing dissolution‐resistant robust interphases to enhance the elevated temperature cycle life of SIBs.

## Graphical abstract

A functional additive of sodium difluorobis(oxalato) phosphate (NaDFBOP) is unprecedentedly synthesized to significantly enhance the cycle life of a commercial NaNi_1/3_Fe_1/3_Mn_1/3_O_2_/hard carbon sodium ion battery at elevated temperatures. NaDFBOP favors the formation of dissolution–resistant robust interphase layers enriched of dissolution resistant oxalate‐containing species and inorganic NaF, which have strong mutual binding energy.

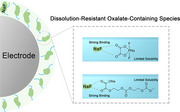

## 1 Introduction

Rechargeable sodium‐ion batteries (SIBs) are viewed as one of the most viable options for large‐scale smart grids and energy storage applications, primarily due to the plentiful availability and affordability of sodium.^[^ ^1^, ^2^ ^]^ Consequently, to improve the performance of SIBs, considerable advancements have been made in developing electrode materials and electrolytes, along with a deeper understanding of their operational and failure mechanisms.^[^ ^3^, ^4^, ^5^, ^6^, ^7^, ^8^, ^9^, ^10^, ^11^, ^12^, ^13^, ^14^, ^15^, ^16^, ^17^ ^]^ However, SIBs consistently exhibit unsatisfactory cycle life at elevated temperatures, significantly hindering their commercial development.^[^ ^18^, ^19^, ^20^ ^]^ On one hand, the solid electrolyte interphase (SEI) layer on the surface of the most available hard carbon (HC) anode is prone to degradation at elevated temperatures because of the high solubility of its components (such as NaF and Na_2_CO_3_) in carbonate‐based electrolytes.^[^ ^12^, ^21^, ^22^, ^23^, ^24^, ^25^ ^]^ This can result in repeated damage and reformation of the SEI layer during cycling, thus leading to severe electrolyte depletion, loss of active sodium, and gas generation. On the other hand, the conventional NaPF_6_‐carbonate based electrolyte is highly susceptible to hydrolysis and thermal decomposition, generating harmful species (e.g., HF, PF_5_, and POF_3_) that can break the cathode electrolyte interphase (CEI) layer, destroy the bulk crystal structure and lead to the dissolution of transition metals.^[^ ^15^, ^26^, ^27^, ^28^ ^]^ Thus, constructing dissolution‐resistant robust SEI/CEI layers is urgently required to improve the cycle life of SIBs, especially at elevated temperatures.

Adjusting the electrolyte components has always been used to build robust SEI/CEI layers that improve the performance of SIBs.^[^ ^11^, ^12^, ^13^, ^14^, ^20^, ^29^, ^30^, ^31^, ^32^, ^33^, ^34^, ^35^, ^36^, ^37^, ^38^, ^39^ ^]^ Wherein, adding small amounts of functional additives to the electrolyte is the most cost‐effective and feasible strategy.^[^ ^11^, ^14^, ^29^, ^31^, ^40^, ^41^, ^42^, ^43^, ^44^, ^45^, ^46^ ^]^ Recently, due to their ability to form robust interphase layers enriched of LiF, a series of lithium fluorophosphates salt‐type additives (such as lithium difluorophosphate (LiDFP), lithium difluorobis(oxalato) phosphate (LiDFBOP), lithium tetrafluoro(oxalato) phosphate (LiOTFP)) have been used to enhance the cycling stability of lithium batteries^[^ ^47^, ^48^, ^49^, ^50^, ^51^, ^52^, ^53^, ^54^, ^55^, ^56^, ^57^, ^58^, ^59^, ^60^, ^61^, ^62^ ^]^ and even sodium‐metal batteries.^[^ ^63^ ^]^ Especially, the LiDFBOP and LiOTFP additive can further polymerize with carbonate solvent to form insoluble protective species on both anodes and cathodes.^[^ ^47^, ^48^, ^49^, ^51^, ^52^, ^55^ ^]^ Consequently, it is expected that the corresponding counterparts of sodium fluorophosphates (e.g., NaDFP, NaDFBOP, NaOTFP) will also contribute to constructing dissolution‐resistant robust SEI/CEI layers for cycle life enhancement of SIBs, especially at elevated temperatures.

In this study, the self‐synthesized sodium difluorobis(oxalato) phosphate (NaDFBOP) additive is unprecedentedly introduced into conventional NaPF_6_‐carbonate based electrolyte to improve the cycle life of the SIB system composed of NaNi_1/3_Fe_1/3_Mn_1/3_O_2_ (NFM) cathode and HC anode, especially at elevated temperatures. With the help of NaDFBOP additive, the NFM/HC full cells deliver a high capacity retention of 85.45% after 1000 cycles at 30 °C and reach 90.76% after 500 cycles at 50 °C. It is revealed that DFBOP⁻ anions enter the first solvation shell of Na⁺, and NaDFBOP possesses lower the lowest unoccupied molecular orbital (LUMO) and higher the highest occupied molecular orbital (HOMO) energy levels, indicative of its high ability to modify SEI/CEI layers. The SEI layer modified with NaDFBOP additive is enriched with inorganic NaF and oxalate‐containing species, thereby demonstrating enhanced mechanical strength. In addition, the dissolution of NaF can be greatly alleviated, which is ascribed to the strong binding ability between dissolution‐resistant oxalate‐containing species and NaF. As for NFM cathode, the particle cracking, bulk crystal structure destruction, and transition metals (TMs) dissolution are significantly suppressed by the NaDFBOP additive. This is closely associated with the alleviated HF generation and formation of dissolution‐resistant robust CEI layer enriched of dissolution‐resistant oxalate‐containing species and NaF. This work underscores the critical importance of designing functional additives and constructing dissolution–resistant robust interphase to enhance cycle life of SIBs at elevated temperatures.

## 2 Results and Discussion

### 2.1 Synthesis and Characterization of NaDFBOP Additive

**Figure 1:**
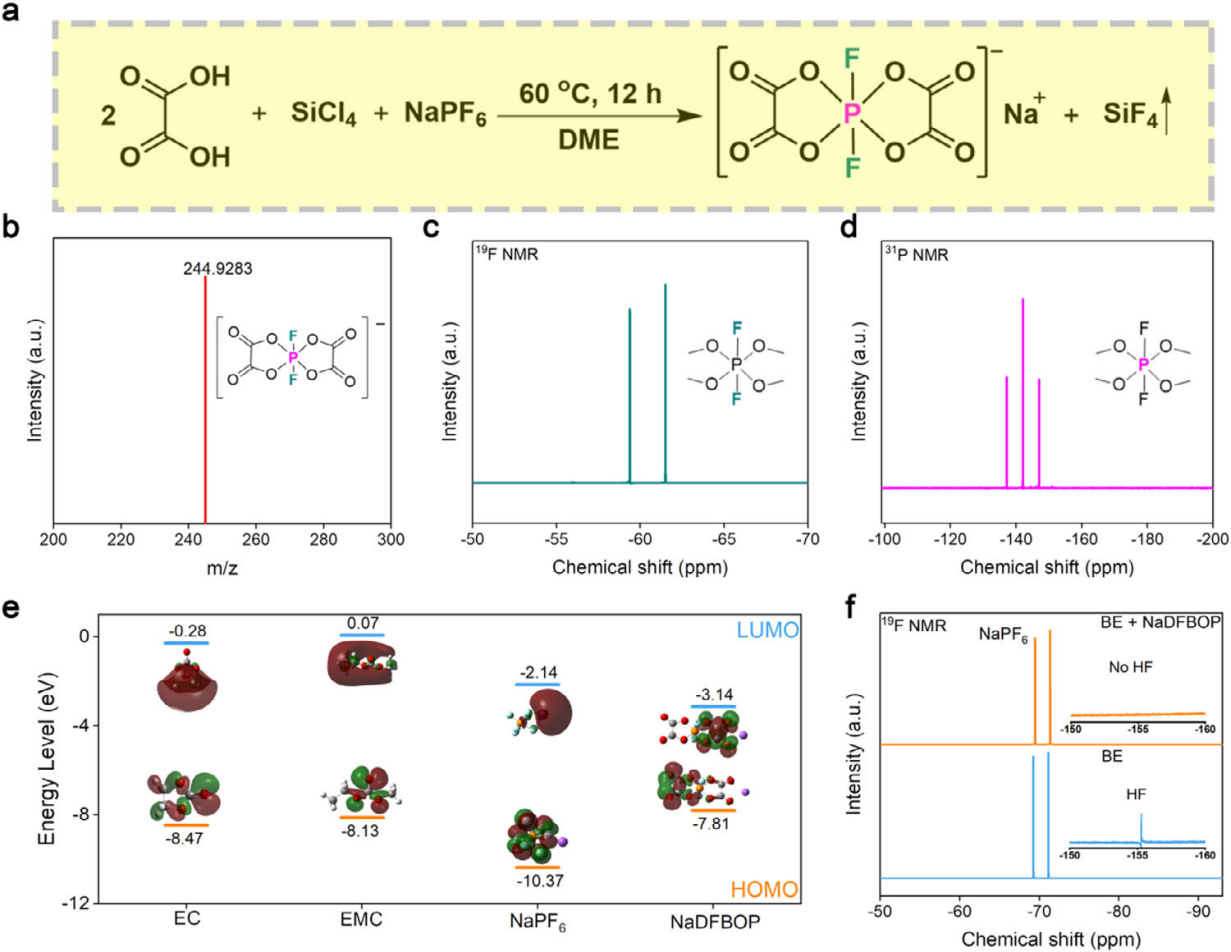
Synthesis and characterization of NaDFBOP. a) Synthetic route of NaDFBOP. b) HRMS of DFBOP⁻ anion in negative ion mode. c) ^19^F NMR spectrum of NaDFBOP. d) ^31^P NMR spectrum of NaDFBOP. e) HOMO and LUMO energy levels of EC, EMC, NaPF_6_, and NaDFBOP. f)^19^F NMR spectrum of the cycled electrolyte extracted from the cycled NFM/HC full cells.

The NaDFBOP compound is successfully synthesized using NaPF_6_, oxalic acid, and tetrachlorosilane as starting materials through a one‐step, one‐pot technique (**Figure** **1**a). As shown in Figure 1b, the negative ion mode of high‐resolution mass spectrum (HRMS) results show a prominent peak at *m/z* = 244.9283 corresponding to the DFBOP⁻ anion. The successful synthesis of NaDFBOP is also confirmed by nuclear magnetic resonance (NMR) spectroscopy (^19^F NMR and ^31^P NMR). In ^19^F NMR spectrum (Figure 1c), the two sets of peaks at −59.38 and −61.50 ppm result from the splitting of the phosphorus (P) element in a chemical environment with two fluorine (F) species. As for ^31^P NMR spectrum (Figure 1d), the triple peak centered at −142.06 ppm indicates the presence of two monofluorine groups attached to the phosphorus (P) atom. In general, density functional theory (DFT) calculation of LUMO and HOMO energy levels are crucial parameters for preliminarily evaluating the reductive and oxidative stability of electrolyte components.^[^ ^64^, ^65^, ^66^ ^]^ With its lowest LUMO energy level (−3.14 eV) and highest HOMO energy level (−7.81 eV), the NaDFBOP additive exhibits a tendency to preferentially undergo reduction at HC anode and oxidation at the NFM cathode, showing its strong capability to participate in the formation SEI and CEI layers (Figure 1e). Regarding functional additives in PF_6_⁻ anion based electrolytes, it is necessary to evaluate their capability of scavenging HF and inhibiting PF_6_⁻ hydrolysis. In this manuscript, the used baseline electrolyte (BE) is 1 M NaPF_6_ EC/EMC = 3/7 by volume. The cycled electrolytes (BE and BE + 1 wt.% NaDFBOP) extracted from the cycled NFM/HC full cells are tested by ^19^F NMR. In Figure 1f, the pronounced doublet peaks at −69 and −71 ppm corresponded to PF_6_⁻ anion. Obviously, the characteristic HF peak at −155.3 ppm disappears with the assistance of the NaDFBOP additive.

### 2.2 The Effects of NaDFBOP Additive on Performances of NFM/HC Full Cells

**Figure 2:**
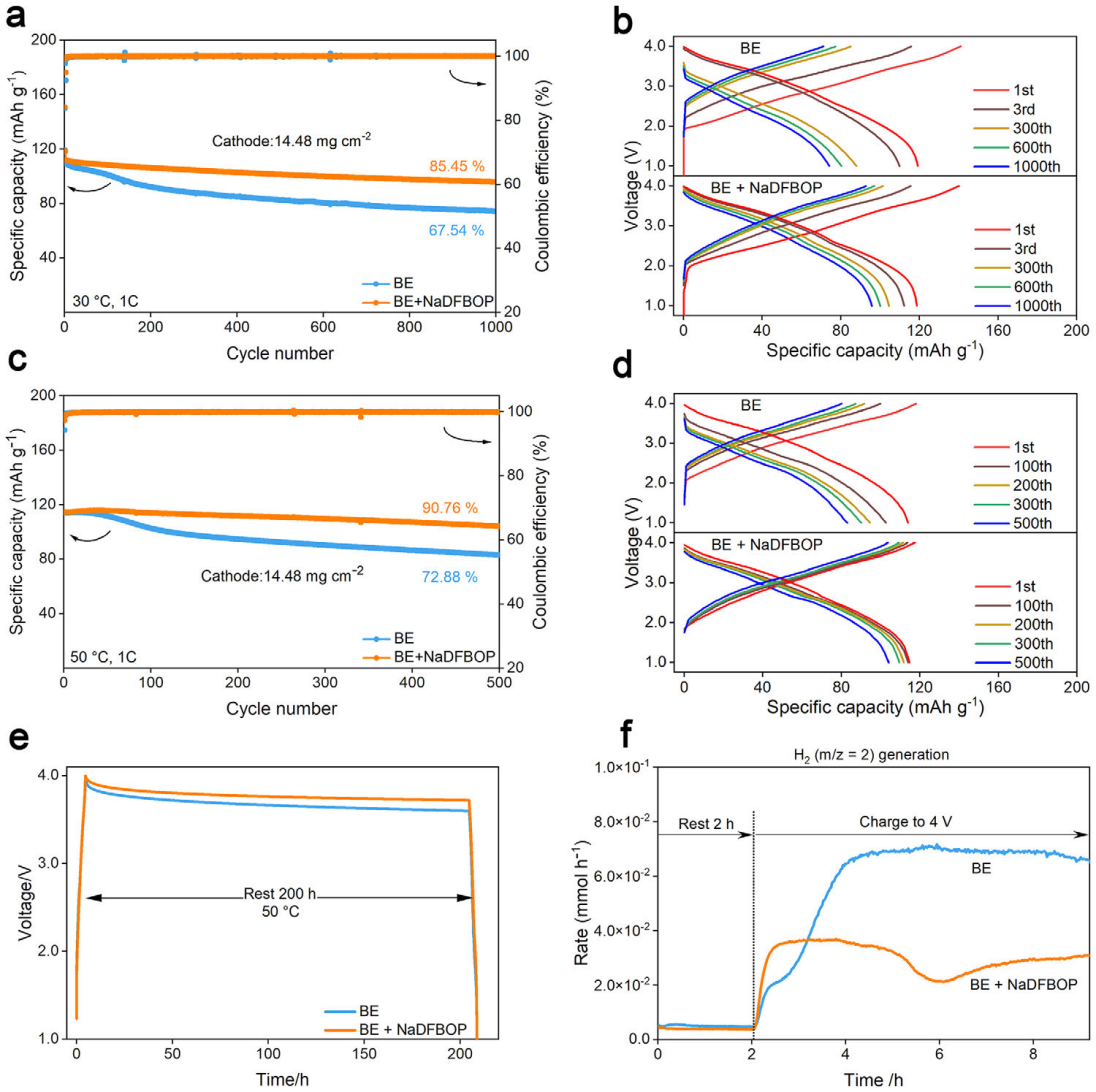
The effect of NaDFBOP additive on performances of NFM/HC full cells. a,b) Room temperature (30 ℃) cycling performance of NFM/HC full cells using BE and BE + NaDFBOP at 1C after tow formation cycles at 0.2 C. c,d) Elevated temperature (50 ℃) cycling performance of NFM/HC full cells using BE and BE + NDFBOP at 1C. e) Self‐discharge tests are conducted on NFM/HC full cells assembled with BE and BE + NaDFBOP electrolytes. The cells are first charged to 4 V at 0.2 C and then left to rest at 50 °C. f) In situ DEMS signals (*m/z* = 2, H_2_) of NFM/HC full cells during the initial charge process using BE and BE + NaDFBOP.

Then, the significant impact of the NaDFBOP additive on the electrochemical performances of NFM/HC full cells (1–4 V, NFM loading 14.48 mg cm^−2^, HC loading 7.4 mg cm^−2^) is systematically investigated. The optimized NaDFBOP addition into BE is determined to be 1 wt.% (Figures S1, S2, Supporting Information). Thus, the following comparisons will be conducted between BE and BE + 1 wt.% NaDFBOP. At room temperature, the NFM/HC full cells utilizing BE exhibit a low capacity retention of merely 67.54% (74.17 mAh g^−1^/109.82 mAh g^−1^, average Coulombic efficiency 99.92%) at a 1 C rate after 1000 cycles (**Figure** **2**a,b). With the help of the NaDFBOP additive, the capacity retention is increased to 85.45% (95.82 mAh g^−1^/112.14 mAh g^−1^, average Coulombic efficiency 99.98%). In subsequent, the electrochemical impedance spectroscopy (EIS) measurements revealed that incorporating NaDFBOP additive into the electrolyte substantially decreased the cell resistance (Figure S3, Supporting Information). Encouragingly at an elevated temperature of 50 °C for 500 cycles, the capacity retention of NFM/HC full cells dramatically increases from 72.88% (83.11 mAh g^−1^/114.03 mAh g^−1^, average Coulombic efficiency 99.88%) to 90.76% (104.1 mAh g^−1^/114.7 mAh g^−1^, average Coulombic efficiency 99.89%) by NaDFBOP additive (Figure 2c,d). To the best of knowledge, the NaDFBOP additive endows the NFM/HC SIBs using NaPF_6_‐carbonate electrolyte with superior cycling stability at elevated temperature (refer to Table S1, Supporting Information), highlighting the critical importance of designing effective additives. The rate capability, which refers to the ability of a battery to maintain its capacity and voltage stability under varied charge‐discharge current rates, stands as a critical parameter for evaluating battery performance.^[^ ^67^ ^]^ Then, the NFM/HC full cells employing BE + NaDFBOP electrolyte exhibit superior rate capability, demonstrating a remarkable discharge capacity of 97.85 mAh g^−1^ even under high‐rate operation at 5 C (Figure S4, Supporting Information). Moreover, the storage performances of fully charged NFM/HC full cells at both room temperature (Figure S5, Supporting Information) and 50 °C (Figure 2e) are improved by NaDFBOP additive. Subsequently, the gas evolution during the first charging process of the NFM/HC full cells system at 30 °C is investigated using in situ differential electrochemical mass spectrometry (DEMS). Obviously, the significant generation of H_2_ (*m/z* = 2) is greatly suppressed by NaDFBOP additive, indicative of the alleviated electrolyte decompositions (Figure 2f). In summary, it is concluded that the improved cycling stability, reduced self‐discharge behavior and suppressed gas evolution of NFM/HC SIBs, particularly at elevated temperatures, are significantly linked to the modification of SEI/CEI layers by the NaDFBOP additive.

### 2.3 The Effects of NaDFBOP Additive on the Electrolyte Solvation Structure

**Figure 3:**
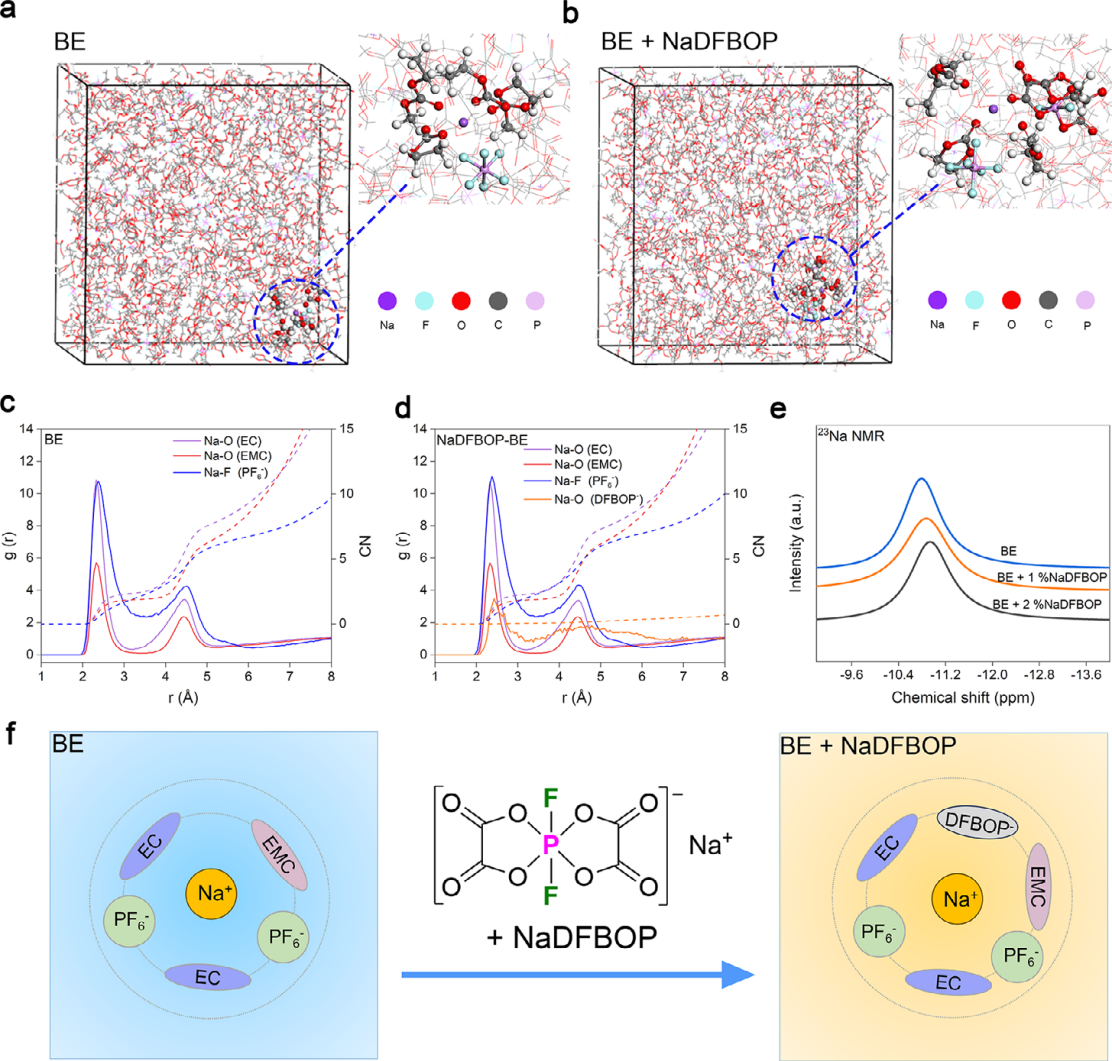
Effects of NaDFBOP additive on electrolyte solvation structure. MD simulation snapshots of a) BE and b) BE + NaDFBOP. Radial distribution function (RDF) curves and coordination numbers curves of Na^+^ in c) BE and d) BE + NaDFBOP. e) ^23^Na spectra of BE and BE with different concentrations of NaDFBOP additive. f) Corresponding schematic illustration of the solvation structures variation when NaDFBOP additive is added.

Electrolyte solvation structure together with the molecular orbital theory (LUMO and HOMO energy level) is always used to assess the capability of functional additives in modifying the SEI/CEI layers.^[^ ^68^ ^]^ As illustrated in Figure 1e, the NaDFBOP additive with lower LUMO energy level and higher HOMO energy level exhibits a tendency to preferentially participate in the formation SEI and CEI layers. According to classic electrolyte solvation chemistry, the enrichment of anions in the first solvation shell of cations will easily contribute to the modification of SEI/CEI layers by forming robust inorganic species.^[^ ^13^, ^69^, ^70^ ^]^ Thus, the effects of the NaDFBOP additive on the solvation structure of NaPF_6_‐carbonate based electrolytes are further evaluated through molecular dynamics (MD) simulations.^[^ ^71^ ^]^ The snapshots (**Figure** **3**a,b) and radial distribution functions (RDFs, Figure 3c,d) reveal that the incorporation of the NaDFBOP additive alters the solvation structure of the carbonate electrolyte. The RDF of BE indicates that the prominent peaks of solvent molecules (EC/EMC) and PF_6_⁻ anions are located at 2.33 and 2.38 Å, respectively, within the solvation shell of Na^+^. With the addition of the NaDFBOP additive, new peak values (2.43 Å) representing Na⁺‐DFBOP⁻ interactions emerged, indicating that DFBOP⁻ anions enter the first solvation shell of Na^+^. Although the average coordination number of DFBOP⁻ with is relatively low at 0.10 due to its low concentration, it exhibits a notable capability to participate in the first Na^+^ solvation shell. We further investigate the impact of NaDFBOP introduction on the solvation behavior of the electrolyte using NMR. The ^23^Na NMR signal will shift up‐field (more negative), indicating an increase in the electron cloud density around ^23^Na. This increase in electron density may be influenced by the surrounding solvent or anion. As shown in Figure 3e, the ^23^Na chemical shift (−10.88 ppm) in BE containing 1% NaDFBOP is more negative than that in BE (−10.77 ppm), indicating a strong interaction between the DFBOP⁻ anion and Na^+^. With the increase in the NaDFBOP addition ratio from 1% to 2%, the negative chemical shift in ^23^Na NMR (−10.96 ppm) further confirms the presence of strong interactions between DFBOP⁻ anions and Na^+^ cations.^[^ ^72^, ^73^ ^]^

The strong coordination ability of DFBOP⁻ arises from its two electron‐rich oxygen atoms, enabling it to effectively compete with EC and EMC molecules in coordinating with Na^+^. The existence of DFBOP⁻ anions in the first solvation shell of Na^+^ is illustrated in Figure 3f.

### 2.4 Characterization of SEI Layer on HC Anode

**Figure 4:**
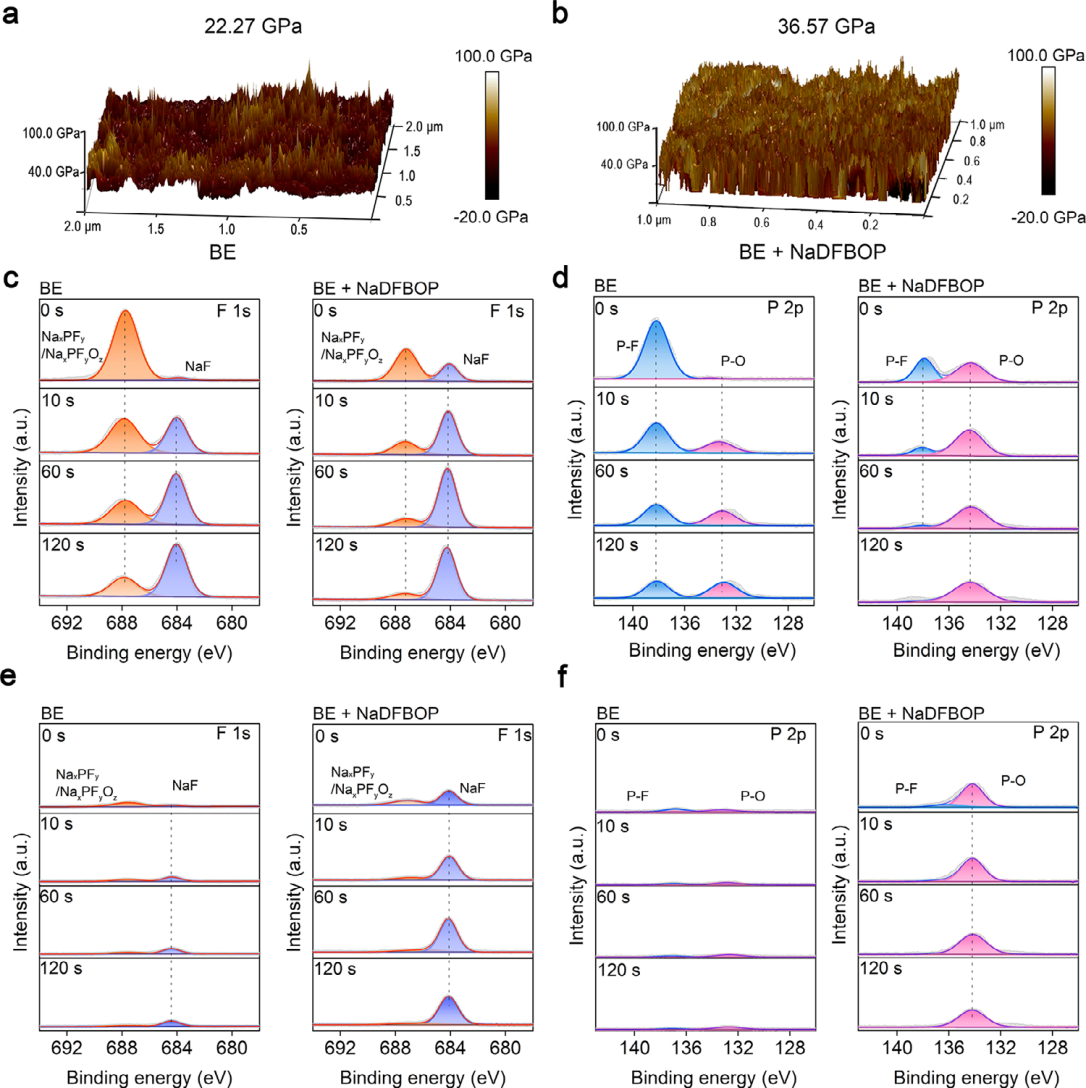
SEI layer characterizations of the cycled HC anode. Surface Young's modulus mappings of the HC anode cycled in a) BE and b) BE + NaDFBOP. c) F 1s and d) P 2p depth‐profiling XPS spectra of the HC anode cycled in BE and BE + NaDFBOP. e) F 1s and (f) P 2p depth‐profiling XPS spectra of the cycled‐soaked HC anode.

Subsequently, the effects of NaDFBOP additive on the SEI layer of HC anode are investigated. From the atomic force microscope (**Figure** **4**a,b), the SEI layer modified by NaDFBOP additive possesses a high Young's modulus (36.57 GPa) compared to the BE counterpart (22.27 GPa). This indicates the enrichment of inorganic species in the additive modified SEI layer.

**Figure 5:**
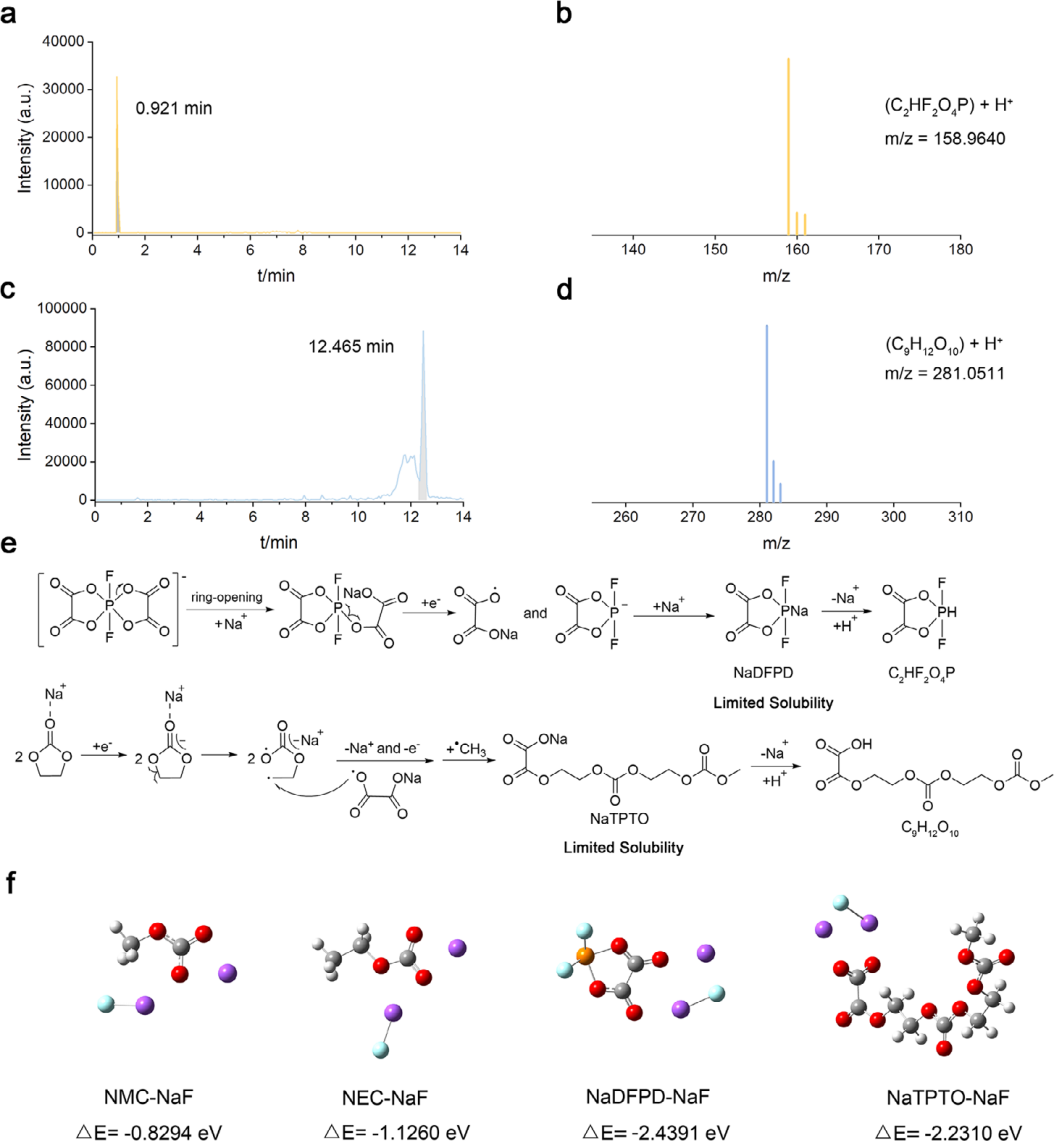
Identification of dissolution‐resistant oxalate‐containing species within SEI layer of HC anode. The related fragment ions and their corresponding *m/z* ratios were detected in the LC‐QTOF‐MS experiments. Liquid chromatography results in the leaching solution of the HC anode, with chromatographic retention times of a) 0.921 min and c) 12.465 min. The *m/z* ratios of the corresponding molecular structures, b) C_2_HF_2_O_4_P and d) C_9_H_12_O_10_, have been annotated in the mass spectra. The distinguishing peaks within mass spectra are observed in positive ion mode. During the liquid chromatography (LC) separation process utilizing 0.1% aqueous formic acid and methanol solution, NaDFPD and NaTPTO transform C_2_HF_2_O_4_P and C_9_H_12_O_10_ by releasing Na^+^ and accepting H^+^. e) The possible formation mechanisms of dissolution‐resistant oxalate‐containing species from NaDFBOP additive. The limited solubility of oxalate‐containing species contributes to the dissolution‐resistant of the NaDFBOP‐derived SEI layer. f) The binding energy of NaF with NMC, NEC, NaDFPD, and NaTPTO.

Electron paramagnetic resonance (EPR) is always used to determine the formation of Na clusters in HC anode.^[^ ^15^ ^]^ It is revealed by EPR that NaDFBOP additive effectively promotes the formation of quasi‐metallic sodium clusters in HC anode (Figure S6, Supporting Information). Then, in‐depth X‐ray photoelectron spectroscopy (XPS) is applied to characterize the chemical compositions of SEI layer on HC anode after cycling. The fitted XPS profiles of F 1s and P 2p are shown in Figure 4c,d, respectively. It is inferred that the enrichment of NaF (684.1 eV, F 1s)^[^ ^42^ ^]^ contributes to increasing the Young's modulus of SEI layer (Figure 4c). Obviously, the formation of NaF and P−O containing species (134.2 eV, P 2p, Figure 4d) from NaDFBOP additive are enhanced and the generation of Na_x_PF_y_O_z_/Na_x_PF_y_ (687.2 eV, Figure 4c) and P−F (137.8 eV, P 2p, Figure 4d) from NaPF_6_ decomposition is alleviated. Moreover, the enhanced C═O containing species is ascribed to the oxalate‐containing species from NaDFBOP additive (Figure S7, Supporting Information). To further investigate the robustness of the NaDFBOP additive modified SEI layer, the cycled HC anodes are immersed in EC/EMC mixtures for 24 h. The SEI components (NaF, P−O species, Na_x_PF_y_O_z_/Na_x_PF_y_, P−F) formed in BE are highly soluble in carbonate solvents, causing them to almost completely disappear (Figure 4e,f). Despite the complete dissolution of Na_x_PF_y_O_z_/Na_x_PF_y_ and P−F species, the NaF and P−O species are well preserved in the NaDFBOP‐modified SEI layer. This suggests a high binding ability of highly soluble NaF to NaDFBOP additive derived reduction products. It was previously pointed out that dissolution‐resistant oxalate‐containing species possess higher binding with inorganic species compared to carbonate‐derived interphase species.^[^ ^74^ ^]^ To gain a deeper understanding of the SEI components derived from NaDFBOP additive, liquid chromatography‐quadrupole time‐of‐flight mass spectrometry (LC‐QTOF‐MS) is conducted. Two representative dissolution‐resistant oxalate‐containing species (NaDFPD, sodium 2,2‐difluoro‐1,3,2λ^5^‐dioxaphospholane‐4,5‐dione; NaTPTO, Sodium 3,8,13‐trioxo‐2,4,7,9,12‐pentaoxatetradecan‐14‐oate) are illustrated. Owing to the liquid chromatography (LC) separation process utilizing 0.1% aqueous formic acid and methanol solution, NaDFPD and NaTPTO transform C_2_HF_2_O_4_P and C_9_H_12_O_10_ by releasing Na^+^ and accepting H^+^ (m/z = 158.9640, (C_2_HF_2_O_4_P + H^+^) (**Figure** **5**a,b); m/z = 281.0511, (C_9_H_12_O_10_ + H^+^) (Figure 5c,d)) The possible formation mechanisms are illustrated in Figure 5e. Owing to the lowest binding energy of P−O^[^ ^56^ ^]^, the DFBOP⁻ anion gains one electron and undergoes homolytic cleavage of the P−O, resulting in the formation of the anionic C_2_F_2_O_4_P^−^ and the radical NaC_2_O_4_•. The C_2_F_2_O_4_P^−^ anionic accepted one Na^+^ toward the fragment of P−O containing species (NaDFPD). On the other hand, NaC_2_O_4_• reacts with the ring‐opening radical product of EC,^[^ ^75^ ^]^ resulting in the formation of EC‐derived polyanionic oligomers with terminal groups substituted by oxalic acid (NaTPTO). It is also inferred that fragments of NaDFPD and NaTPTO may also originate from one large‐molecular dissolution‐resistant oxalate‐containing species. As shown in Figure 5f, through theoretical calculations, we comparatively investigated the binding energies between NaF and representative carbonate‐derived species (e.g. NMC, Sodium methyl carbonate; NEC, Sodium ethyl carbonate), and the binding energies between NaF and oxalate‐containing species (e.g. NaDFPD, NaTPTO). The results demonstrate that oxalate‐containing species exhibit significantly enhanced binding energy with NaF compared to carbonate‐containing species. Thus, the dissolution of NaF is greatly suppressed by oxalate‐containing species. In summary, the NaDFBOP additive favors the formation of a robust SEI layer enriched of dissolution‐resistant oxalate‐containing species and inorganic NaF, which have strong mutual binding energy (Figure S8, Supporting Information).

### 2.5 Characterization of Cycled NFM Cathode at Elevated Temperature

**Figure 6:**
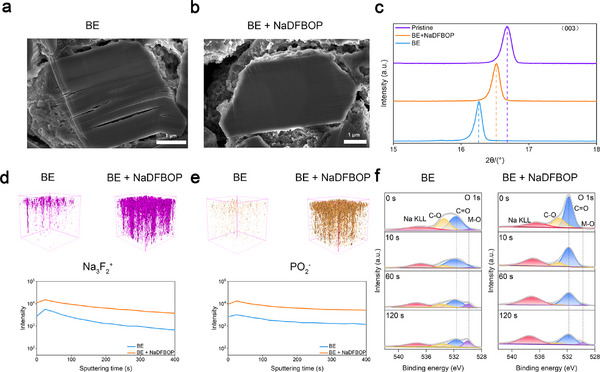
Effects of NaDFBOP additive on cycled NFM cathode at elevated temperatures. Cross‐sectional SEM images of cycled NFM cathode in a) BE and b) BE + NaDFBOP. c) XRD patterns of cycled NFM cathode in BE and BE + NaDFBOP. d) Na_3_F_2_^+^ and e) PO_2_⁻ TOF‐SIMS 3D reconstruction and corresponding depth profiles of cycled NFM cathode in BE and BE + NaDFBOP. f) O 1s depth‐profiling XPS spectra of the cycled NFM cathode in BE and BE + NaDFBOP. The NFM/HC full cells are cycled at 50 °C for 100 cycles.

Finally, the effects of NaDFBOP additive on the cycled NFM cathode at 50 °C are examined. High‐resolution transmission electron microscopy (HRTEM) images reveal that the NaDFBOP additive favors the formation of a uniform and thick CEI layer on the elevated temperature cycled NFM cathode (Figure S9, Supporting Information). Obviously from cross‐sectional scanning electron microscope (SEM), the severe microcracking of elevated temperature cycled NFM cathode is greatly suppressed by this unique CEI layer (**Figure** **6**a,b). Moreover, inductively coupled plasma optical emission spectrometry (ICP‐OES) testing indicates that the dissolution of transition metal ions, particularly Fe ions, from fully charged NFM cathode can be significantly reduced by NaDFBOP additive (Figure S10, Supporting Information). The XRD pattern also revealed that the bulk crystal structure of NFM cathode is better preserved when cycled in BE + NaDFBOP (Figure 6c), also confirming the protective effect of the as‐formed CEI layer. From the time‐of‐flight secondary ion mass spectrometry (TOF‐SIMS) 3D element reconstruction image of NFM cathode, one can note that large amounts of Na_3_F_2_⁺ (indicative of NaF, Figure 6d) and PO_2_⁻ (indicative of P−O containing species, Figure 6e) are distributed through the CEI layer after the addition of NaDFBOP. From O 1s in‐depth XPS, the increased C═O (531.7 eV) signal suggests that the formed CEI layer favored by NaDFBOP is enriched of oxalate‐containing species (Figure 6f). To gain a deeper understanding of the CEI components derived from NaDFBOP additive, LC‐QTOF‐MS is also conducted. Two representative dissolution‐resistant oxalate‐containing species of NaDFPD (P−O containing species, Figure S11, Supporting Information) and NaTPTO (oligomers, Figure S12, Supporting Information) from NaDFBOP additive are detected. It was previously pointed out that dissolution‐resistant oxalate‐containing species possess higher binding with inorganic species compared to carbonate‐derived interphase species.^[^ ^74^ ^]^ Thus, the NaDFBOP additive also favors the formation of a robust CEI layer enriched of dissolution‐resistant oxalate‐containing species and inorganic NaF, which have strong mutual binding energy (Figure S13, Supporting Information).

## 3 Conclusion

In this study, NaDFBOP additive is synthesized and introduced into NaPF_6_‐carbonate electrolytes to significantly enhance the cycle life of a commercial NFM/HC SIBs system, particularly at 50 °C. Theoretical calculations demonstrate that the DFBOP⁻ anions enter the first solvation shell of Na⁺. Furthermore, NaDFBOP exhibits lower LUMO and higher HOMO energy levels, suggesting that it has a strong tendency to decompose and a strong ability to modify the SEI/CEI layer. Characterizations suggest that NaDFBOP favors the formation of dissolution–resistant robust interphase layers enriched of dissolution‐resistant oxalate‐containing species and inorganic NaF, which have strong mutual binding energy. This work underscores the critical importance of designing functional additives and constructing robust interphase to enhance the elevated temperature cycle life of SIBs.

## Conflict of Interest

The authors declare no conflict of interest.

## Supporting information



Supporting Information

## Data Availability

The data that support the findings of this study are available from the corresponding author upon reasonable request.
