# Supplementary material for: Constructing Dissolution–Resistant Interphases for Long‐Life Sodium‐Ion Batteries at Elevated Temperatures
Source: Adv Sci (Weinh). 2025 May 8;12(27):2502860. doi: 10.1002/advs.202502860 (PMC12279246; doi:10.1002/advs.202502860)
Supplement: Supplementary file 1 — Supporting Information [file ADVS-12-2502860-s001.docx]

Supporting Information

**Constructing Dissolution-Resistant Interphases for Long-Life Sodium-Ion Batteries at Elevated Temperatures**

Wenting Deng,^[a,b,c,d]^ Xiaofan Du,^[b,c,d]^ Gaojie Xu,^*[b,c,d]^ Shitao Wang,^*[b,c,d^] Li Du,^[b,c,d]^ Tiantian Dong,^[b,c,d]^ Rongxian Wu,^[b,c,d]^ Chuanchuan Li,^[b,c,d]^ Zhaolin Lv,^[b,c,d]^ Jiangwei Ju, ^[b,c,d]^ Xinhong Zhou,^*[a]^ Guanglei Cui^*[b,c,d]^

^[a]^ W. Deng, Prof. X. Zhou

College of Chemistry and Molecular Engineering, Qingdao University of Science and Technology, Qingdao, 266042 China

E-mail: zhouxinhong@qust.edu.cn

^[b]^ W. Deng, Dr. X. Du, Prof. G. Xu, Dr. S. Wang, L. Du, Dr. T. Dong, R. Wu, Dr. C. Li, Dr. Z. Lv, Dr. J. Ju, Prof. G. Cui
Qingdao Industrial Energy Storage Research Institute, Qingdao Institute of Bioenergy and Bioprocess Technology, Chinese Academy of Science, Qingdao 266101, China

E-mail: cuigl@qibebt.ac.cn; xugj@qibebt.ac.cn; wangst@qibebt.ac.cn

^[c]^ W. Deng, Dr. X. Du, Prof. G. Xu, Dr. S. Wang, L. Du, Dr. T. Dong, R. Wu, Dr. C. Li, Dr. Z. Lv, Dr. J. Ju, Prof. G. Cui

Shandong Energy Institute, Qingdao 266101, China

E-mail: cuigl@qibebt.ac.cn; xugj@qibebt.ac.cn; wangst@qibebt.ac.cn

^[d]^ W. Deng, Dr. X. Du, Prof. G. Xu, Dr. S. Wang, L. Du, Dr. T. Dong, R. Wu, Dr. C. Li, Dr. Z. Lv, Dr. J. Ju, Prof. G. Cui

Qingdao New Energy Shandong Laboratory, Qingdao 266101, China

E-mail: cuigl@qibebt.ac.cn; xugj@qibebt.ac.cn; wangst@qibebt.ac.cn

**Experimental Section**

*1.1. Synthesis of sodium difluorobis(oxalato) phosphate (NaDFBOP)*

Oxalic acid (216.1 mg, 2.4 mmol) and 1,2-Dimethoxyethane (DME, 20 mL) were placed in a 100 mL round-bottom flask under an argon atmosphere. Tetrachlorosilane (186.9 mg, 1.1 mmol) was then added slowly to the oxalic acid solution while maintaining an ice bath, and the mixture was stirred for 1 hour at 0 °C. Following this, the ice bath was removed, and the reaction was allowed to proceed at 60 °C for 5 hours. Subsequently, 1.0 mmol of sodium hexafluorophosphate (NaPF_6_) was added, and the reaction continued at 60 °C for 12 hours in DME solution. Finally, n-hexane was added to induce precipitation, resulting in the formation of the white target product, Sodium difluorobis(oxalato) phosphate (NaDFBOP). ^31^P{^1^H}NMR (162 MHz, DMSO-d6): −137.1 (s), −142.1(s), −146.9(s). ^19^F{^1^H}NMR (376 MHz, DMSO-d6): −59.38 (s), −61.50 (s).

*1.2. Electrode and Electrolytes Preparation*

The cathode consisted of 94.5 wt.% NFM (Jiangsu Xiangying Amperex Technology Limited), 3 wt.% conductive carbon (Super P), and 2.5 wt.% polyvinylidene fluoride (PVDF5130, SOLVAY), while the anode consisted of 94.8 wt.% HC, 2 wt.% Super P, 1.2 wt.% carboxymethyl cellulose (CMC), and 2 wt.% styrene butadiene rubber (SBR). The cathode slurry was coated onto an aluminum foil current collector and subsequently subjected to a drying process under vacuum conditions at 110 °C for a period of 24 hours. The anode slurry was coated onto an aluminum foil current collector and subsequently subjected to a drying process under vacuum conditions at a temperature of 100 °C for a period of 24 hours. For coin-type NaNi_1/3_Fe_1/3_Mn_1/3_O_2_ (NFM) /HC full cells (the N/P ratio = 1.2, the cathode mass loading was 14.48 mg cm^−2^, the anode mass loading was 7.4 mg cm^−2^). For coin-type cells, the electrodes were punched into disks with diameters of 12 mm for the cathode and 14 mm for the anode, respectively. As for electrolyte preparation, the baseline electrolyte (BE) was 1 M NaPF_6_ in EC/EMC (3:7, v/v), purchased from DoDoChem (China). Add 0.5 wt.% NaDFBOP, 1 wt.% NaDFBOP, 1.5 wt.% NaDFBOP, 2 wt.% NaDFBOP in BE as experimental samples. All materials were stored and conducted in a glove box filled with argon gas.

*1.3. Cells assembling and testing*

Cells assembling: 2032 coin-type NaNi_1/3_Fe_1/3_Mn_1/3_O_2_ (NFM)/HC cells were assembled using GF/A separator and the electrolyte prepared above (100 μL) in an Ar-filled glove box. The electrochemical tests of these cells (1.0 – 4.0 V) were implemented using a NEWARE system (CT-4008T-5 V 10 mA, NEWARE Technology Limited (China)) at 30 °C or 50 °C. And the cells were charge/discharge at 0.2 C (1 C = 120 mA g^−1^) in the first two pre-cycles, then held long-cycle rate at 1 C. In the full-cell charge/discharge capability test in an elevated-temperature environment, the cells were pre-cycled for two cycles at a rate of 0.2 C at 30 °C, followed by a long cycle at rate of 1 C at 50 °C. The rate capability testing was conducted with 5 charge-discharge cycles at each of 0.2 C, 0.5 C, 1 C, 2 C, and 5 C; then restored to 0.2 C for 5 cycles; finally switched to 1 C rate for long-term cycling performance test.

*1.4. Characterizations*

Nuclear magnetic resonance (NMR) spectra of the samples were acquired using the Bruker AVANCE III 400 MHz instruments. The cycled electrodes for analysis of the surface chemistry and morphology were obtained from the full cells dis-assembled in a glove box. The equipped mass spectrometer in the DEMS system was HPR-20 (Hiden Analytical Ltd.). The electrochemical cell was directly charged to 4 V. The atomic force microscopy (AFM) system (Bruker Corp., Dimension Icon) was employed to collect the topography and modulus images of the cycled HC anode. Using Ar-XPS (Ar plasma etching X-ray photoelectron spectroscopy, Thermo Scientific ESCA Lab 250Xi) and ToF-SIMS (Time-of-flight secondary ion mass spectrometry, PHI nanoTOF II, ULVAC-PHI.INC) to characterize the interphase components. In the SEI dissolution experiments, the cycled electrodes for XPS were immersed in the carbonate solvents for 24 hours to facilitate the complete dissolution of the soluble species in the SEI layer. All electrodes were sealed in airtight containers within a glovebox to maintain an inert atmosphere and subsequently transferred for further characterization. For the liquid chromatography-quadrupole time-of-flight tandem mass spectrometry (LC-QTOF-MS), the cycled HC anode and NFM cathode were disassembled from NFM/HC full cells (10 cycles) using BE + NaDFBOP, then subjected to the following treatment: First, the samples were rinsed three times with EMC to remove residual electrolyte; Subsequently, the HC anode and NFM cathode were individually immersed in deuterated dimethyl sulfoxide (DMSO-d6) for 24 hours in preparation for LC-QTOF-MS analysis. The LC and QTOF-MS analysis was performed on Agilent 1290 UPLC and Agilent 6550 QTOF system (Agilent Technologies). Reversed-phase chromatography was performed using Agilent Poroshell waters BEH C18 (2.1 mm × 100 mm, 1.7 μm) and a flow rate of 0.3 ml min^⁻1^. The mobile phase gradient consisted of water containing 0.1% formic acid (A) and methanol solution (B). To separate more components, the gradient was set as follows: 0–1 min 95 % A; 1–8 min 95–5 % A; 8–13 min 5 % A; 13–14 min 5–95 % A; 14–15 min 95 % A. The data acquisition was performed on a 6550 QTOF mass spectrometer equipped with a dual electrospray ion (ESI) source. Cross-sectional morphology analysis of the cycled NFM cathodes pre-processed by the IM4000 Ion Milling System, then the cross-sectional morphology of the cycled NFM cathodes were observed using SEM (Scanning electron micro-scope, Hitachi S-4800). XRD (X-ray diffraction, Bruker-AXS, Rigaku Corporation) was used to test the crystal structure changes of electrodes. TEM (FEI Tecnai G2 F20 X-Twin) was used to observe the CEI layer on the NFM cathode surface after cycling. The fully charged NFM cathode cycled in BE and BE + NaDFBOP were washed three times with EMC, and then placed in the corresponding electrolytes and stored at 50 °C for 24 hours to determine the dissolution of TMs by ICP-OES (Inductively coupled plasma optical emission spectrometry, Aglient 7850). The electrochemical impedance spectroscopy (EIS) test was carried out on the BioLogic VMP-300 workstation (frequency range: 7 MHz to 100 mHz; amplitude: 10 mV). Electron paramagnetic resonance (EPR) spectra were collected using a Bruker Emx PLUS spectrometer.

*1.5. Theoretical calculations*

HOMO and LUMO energy

The computations were conducted using the Gaussian 16 software package. All calculations utilized the B3LYP functional^[1]^ in combination with the 6-311G(d) basis set.^[2]^ To enhance the accuracy of the calculations, we employed the DFT-D3 method with BJ-damping^[3]^ to account for the weak interaction.

Molecular dynamic simulations

Molecular dynamic (MD) simulations were performed on the electrolyte mixtures (EC, EMC, NaPF_6_, and NaDFBOP) to observe the structure changes of the electrolyte mixtures. First, the optimized electrolyte molecules were packed in a periodic box to construct the bulk systems, the compositions of simulated electrolytes are given in Table S2. The simulation cells contained and 60 NaPF_6_, 270 EC and 402 EMC, 99 NaPF_6_, 440 EC, 660 EMC, and 6 NaDFBOP, respectively. Subsequently, all mixture systems were equilibrated by NPT (i.e., isothermalisobaric) MD simulations for 5 ns at 298 K and atmospheric pressure, followed by NVT (i.e., isothermal) MD simulations for 10 ns with a 1 fs time step. The temperature was controlled by a Nose-Hoover Langevin (NHL) thermostat, and the pressure was controlled by a Berendsen barostat. ^[4, 5]^ The Ewald scheme^[6, 7]^ and atom-based cutoff method (i.e., a radius of 15.5 Å) were applied to treat electrostatic and van der Waals (vdW) interactions, respectively. And the results are demonstrated in **Table S2**.

Quantum chemical calculations

The computations were conducted using the Gaussian 09 software package. All calculations utilized the B3LYP functional^[1]^ in combination with the 6-311+G(d, p) basis set.^[2]^ To enhance the accuracy of the calculations, we employed the DFT-D3 method with BJ-damping^[3]^ to account for the weak interaction. The binding energies between NaF and solvent were defined as the interaction between NaF and NMC, NEC, NaDFPD and NaTPTO molecule fragments. The binding energy (E_bind_) was calculated according to equation, the expression as follows:

$$E_{bind}= E_{m}-E_{s1}-E_{s2}$$

where E_m_, E_s1_, E_s2_ are the energies of the total energy of the system, the NaF, and the NMC, NEC, NaDFPD and NaTPTO molecule fragments.


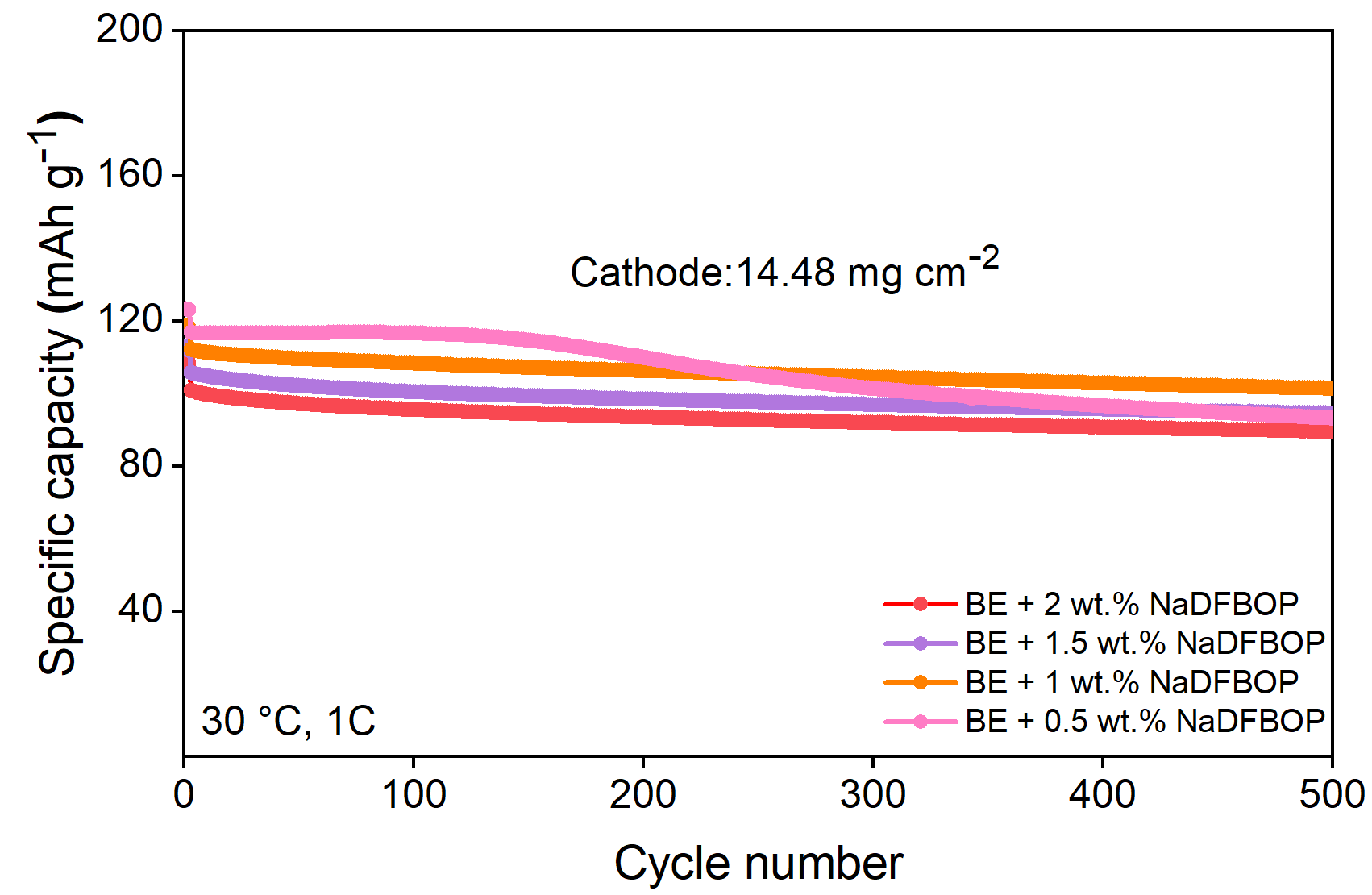


**Figure S1.** The room temperature cycling performance of NFM/HC full cells using BE + 0.5 wt.% NaDFBOP, BE + 1 wt.% NaDFBOP, BE + 1.5 wt.% NaDFBOP, BE + 2 wt.% NaDFBOP.

Considering the delivered specific capacity and capacity retention rate, the NFM/HC full cells using BE + 1 wt.% NaDFBOP demonstrate superior cycle life at 1 C rate.


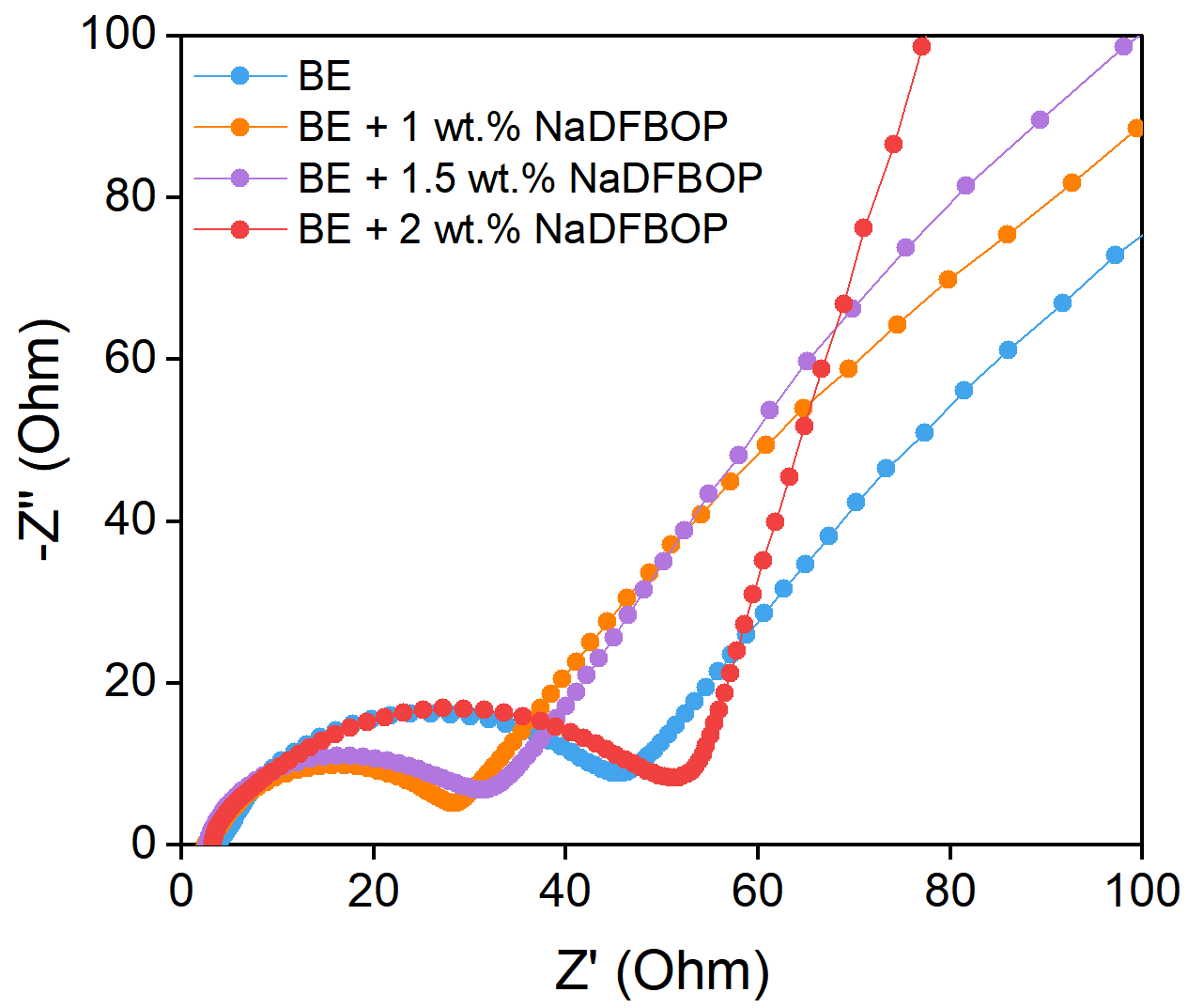


**Figure S2.** EIS of the NFM/HC full cells using BE with different concentrations of NaDFBOP.

A comparison of the EIS spectra of full cells using BE containing different concentrations of NaDFBOP after 10 cycles demonstrated that the resistance of the full cell increased as the additive concentration continued to increase.


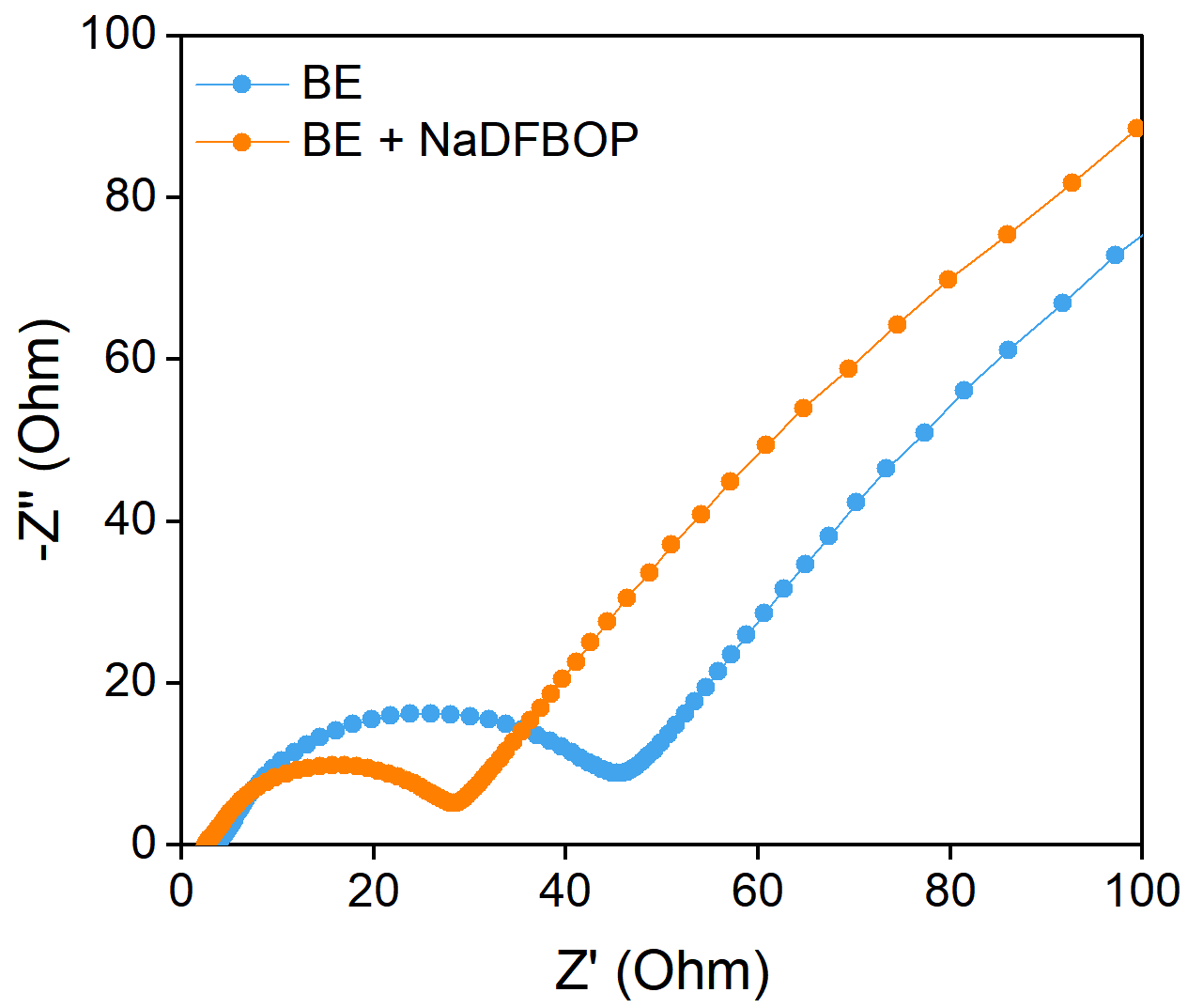


**Figure S3**. EIS of the NFM/HC full cells using BE and BE + NaDFBOP.

The electrochemical impedance spectroscopy (EIS) measurements revealed that incorporating NaDFBOP additive into the electrolyte substantially decreased the cell resistance.


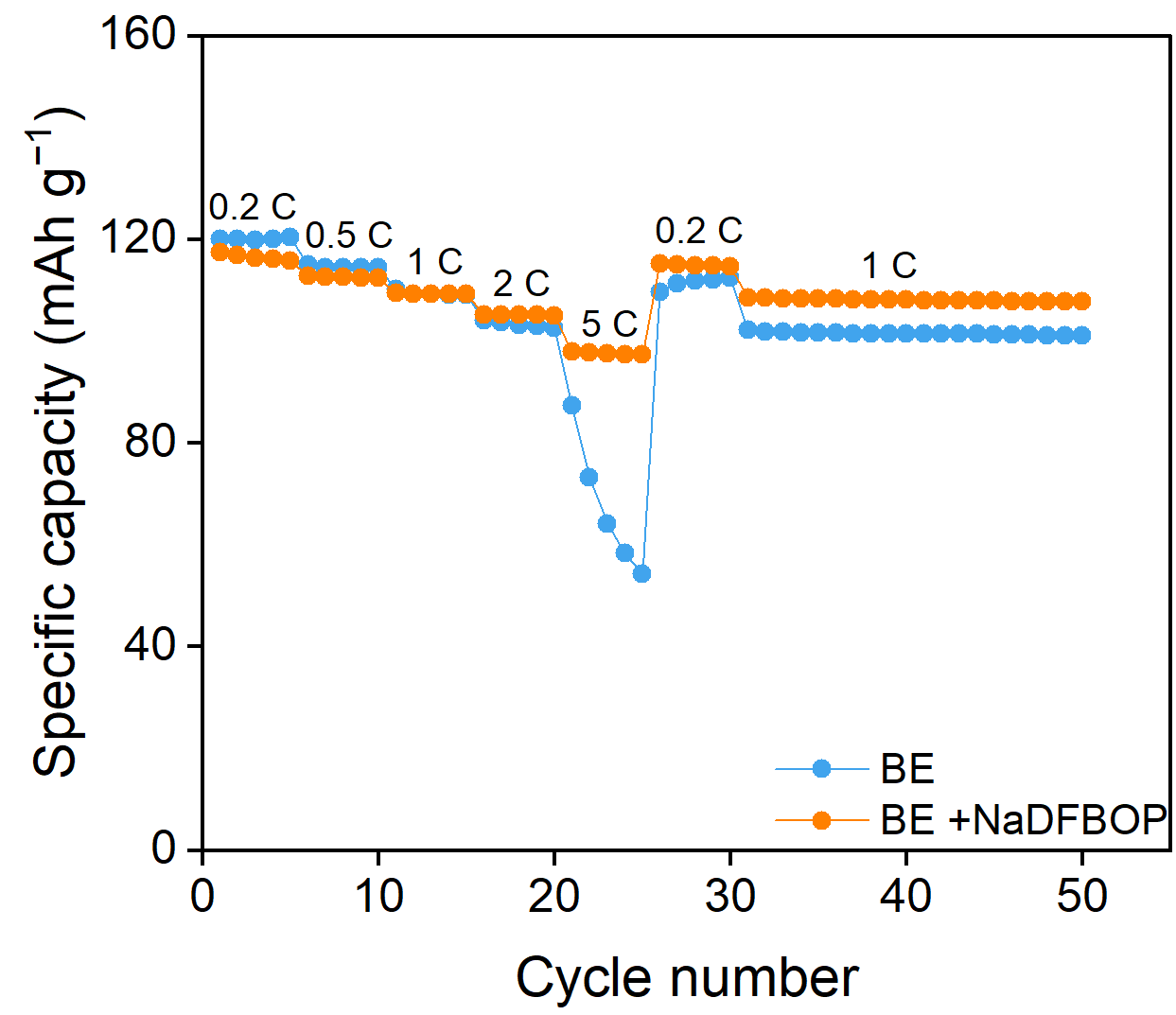


**Figure S4.** Rate capability of the NFM/HC full cells using BE and BE + NaDFBOP.

The NFM/HC full cells employing BE + NaDFBOP electrolyte exhibit superior rate capability, demonstrating a remarkable discharge capacity of 97.85 mAh g^−1^ even under high-rate operation at 5 C.


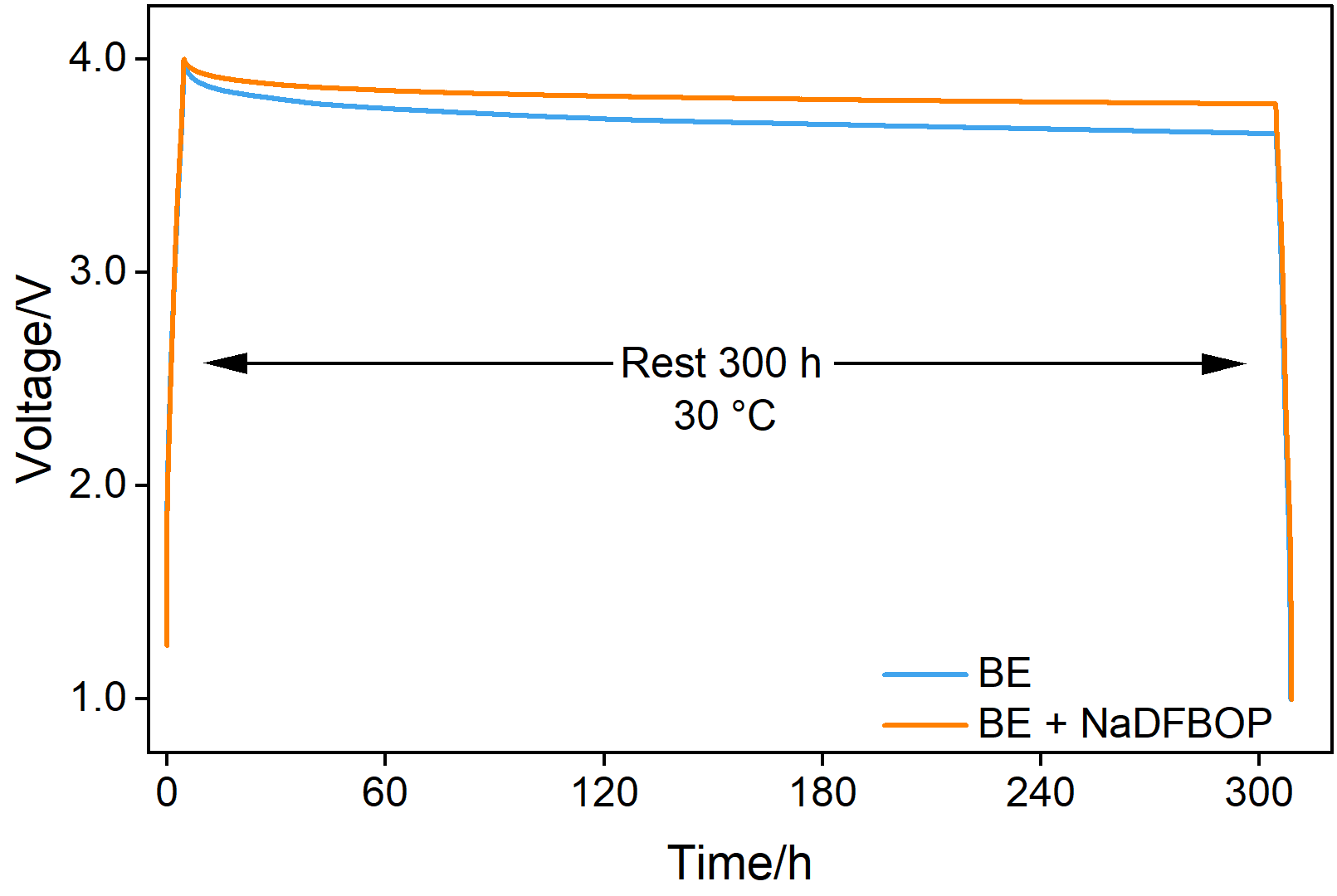


**Figure S5.** The storage performances of NFM/HC full cells using BE and BE + 1 wt.% NaDFBOP at 30 °C.

After being fully charged to 4 V and stored at room temperature for 300 hours, the voltage of the NFM/HC full cells using BE + 1 wt.% NaDFBOP showed a slight decrease from 4.0 V to 3.79 V, indicating minimal self-discharge. In contrast, the voltage of the NFM/HC full cells using the BE dropped significantly to 3.65 V.


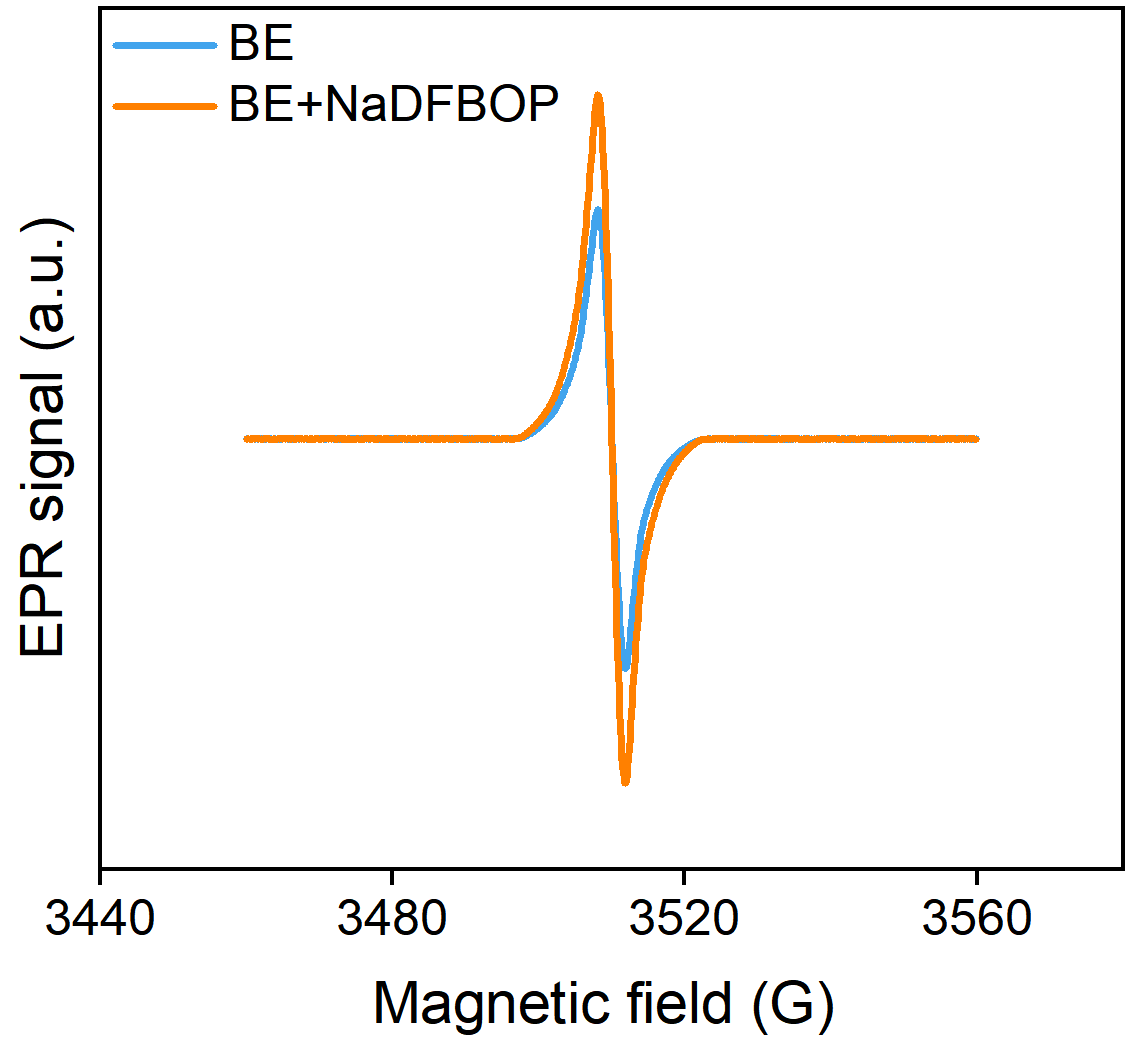


**Figure S6.** EPR spectra of fully sodiated HC anode in BE and BE + NaDFBOP.

Electron paramagnetic resonance (EPR) is always used to determine the formation of Na clusters in HC anode. It is revealed by EPR that NaDFBOP additive effectively promotes the formation of quasi-metallic sodium clusters in HC anode.


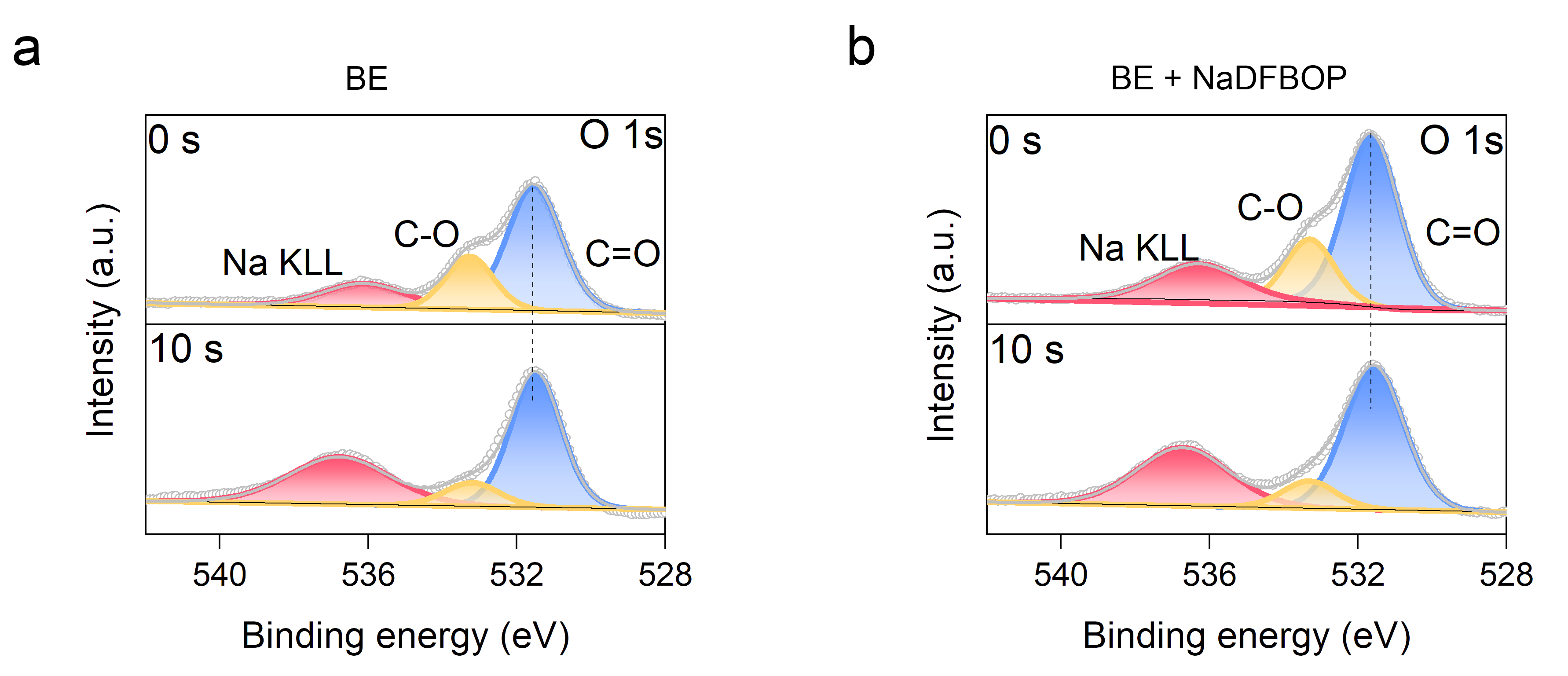


**Figure S7.** O 1s depth-profiling XPS spectra of the cycled HC anode in (a) BE and (b) BE + NaDFBOP.


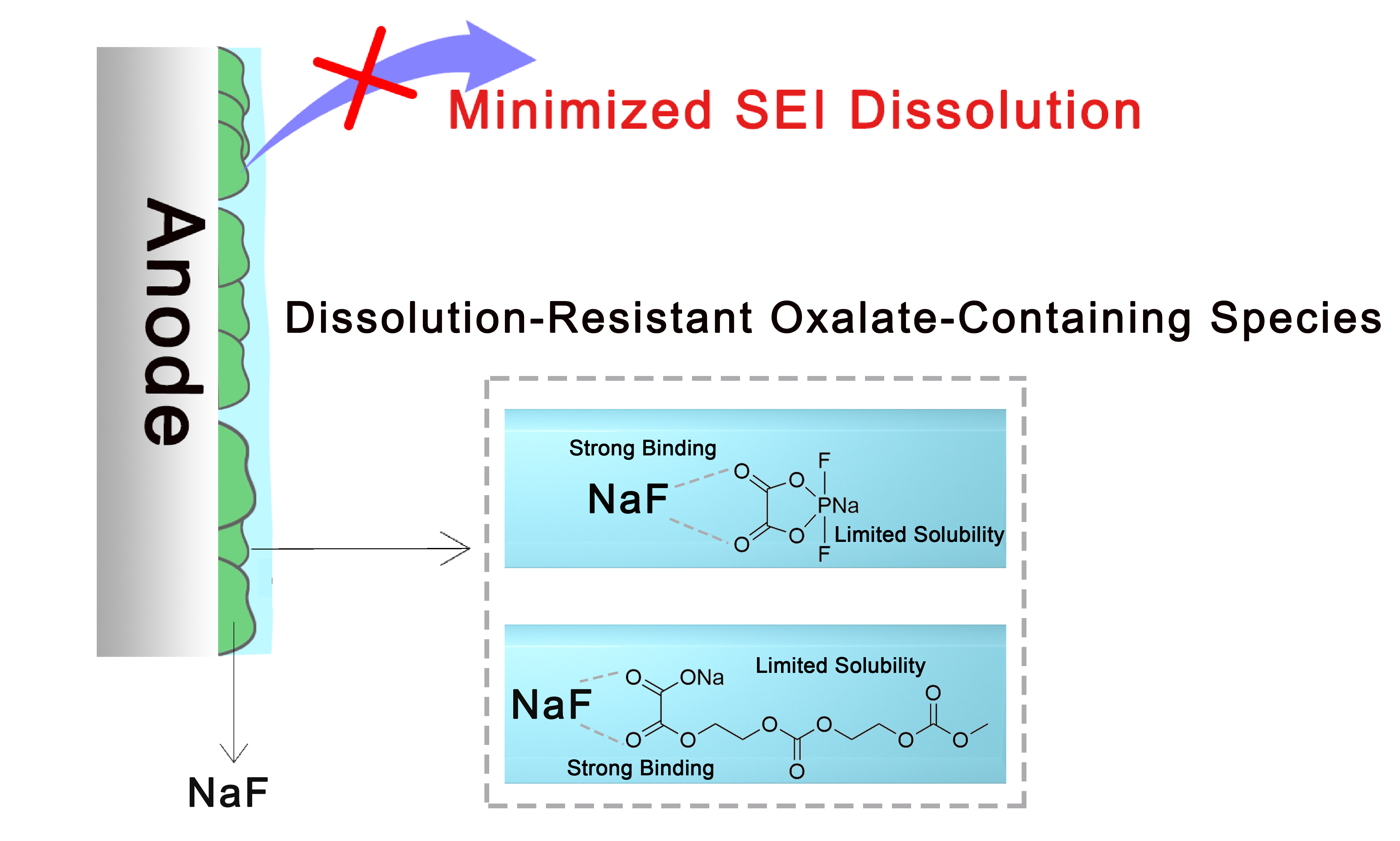


**Figure S8.** Illustration of the formation of a dissolution-resistant robust SEI layer on HC anode by the NaDFBOP additive.

The NaDFBOP additive favors the formation of a robust SEI layer enriched of dissolution-resistant oxalate-containing species and inorganic NaF, which have strong mutual binding energy.


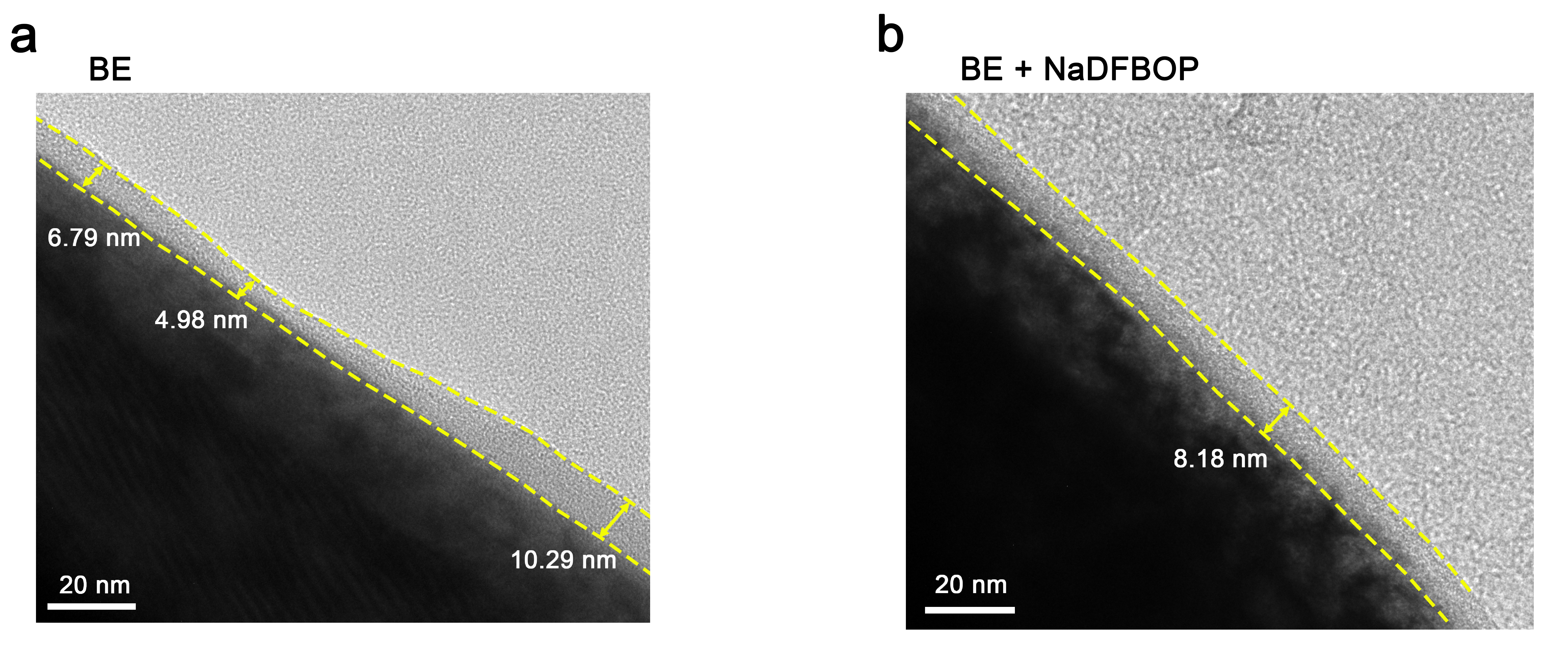


**Figure S9.** HRTEM images of NFM cycled in BE (a) and BE + NaDFBOP (b) at 50 °C for 100 cycles.

High-resolution transmission electron microscopy (HRTEM) images reveal that the cathode electrolyte interphase (CEI) layer formed in the BE is uneven. In contrast, with the help of the NaDFBOP additive, the surface of the NFM cathode is uniformly covered by a compact and homogeneous CEI layer.

**
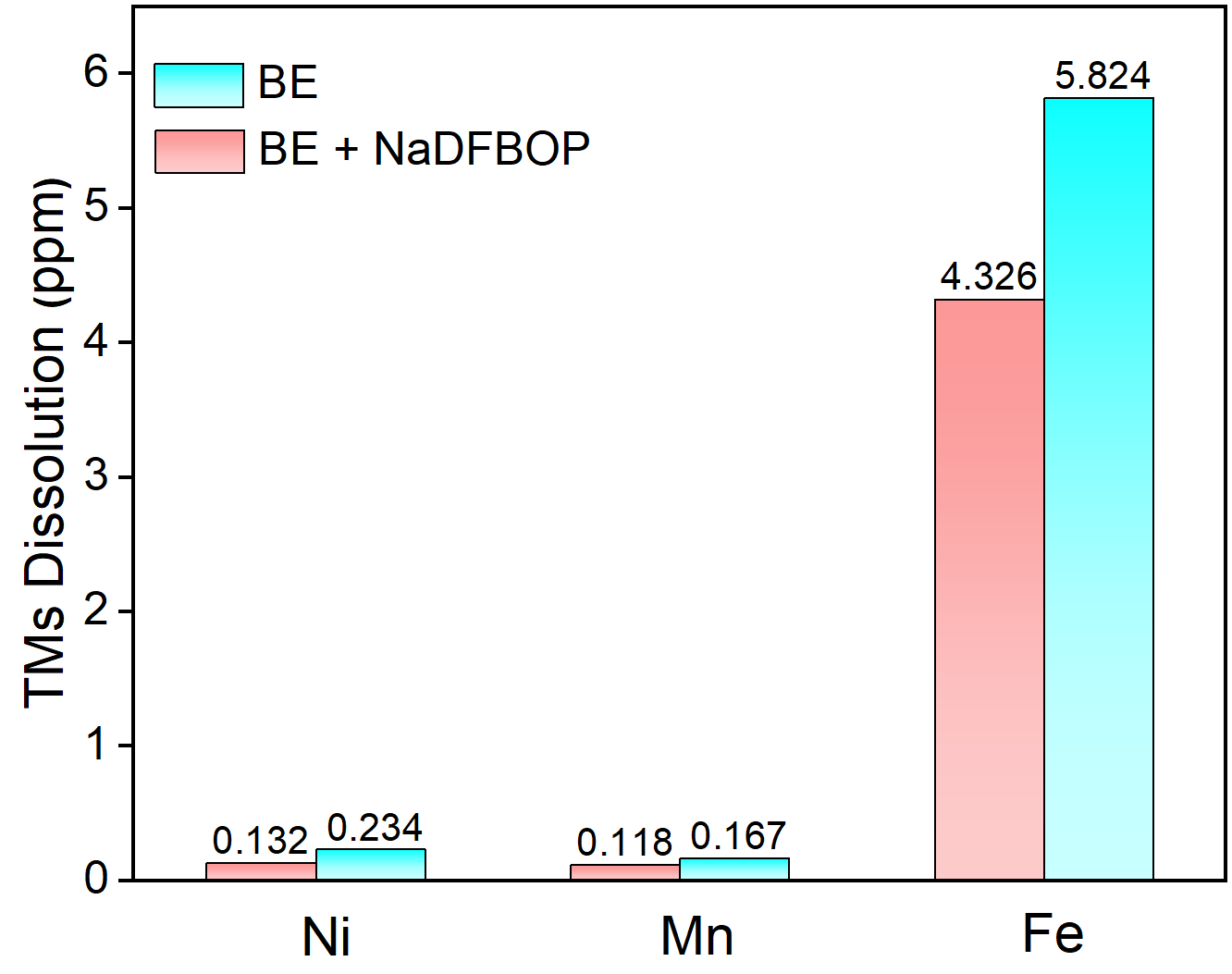
Figure S10.** The dissolution of transition metal ions from NFM cathodes fully charged in different electrolytes.

Inductively coupled plasma optical emission spectroscopy (ICP-OES) tests showed that the dissolution of transition metal ions, especially Fe ions, from the fully charged NFM cathode is significantly reduced by NaDFBOP additive.


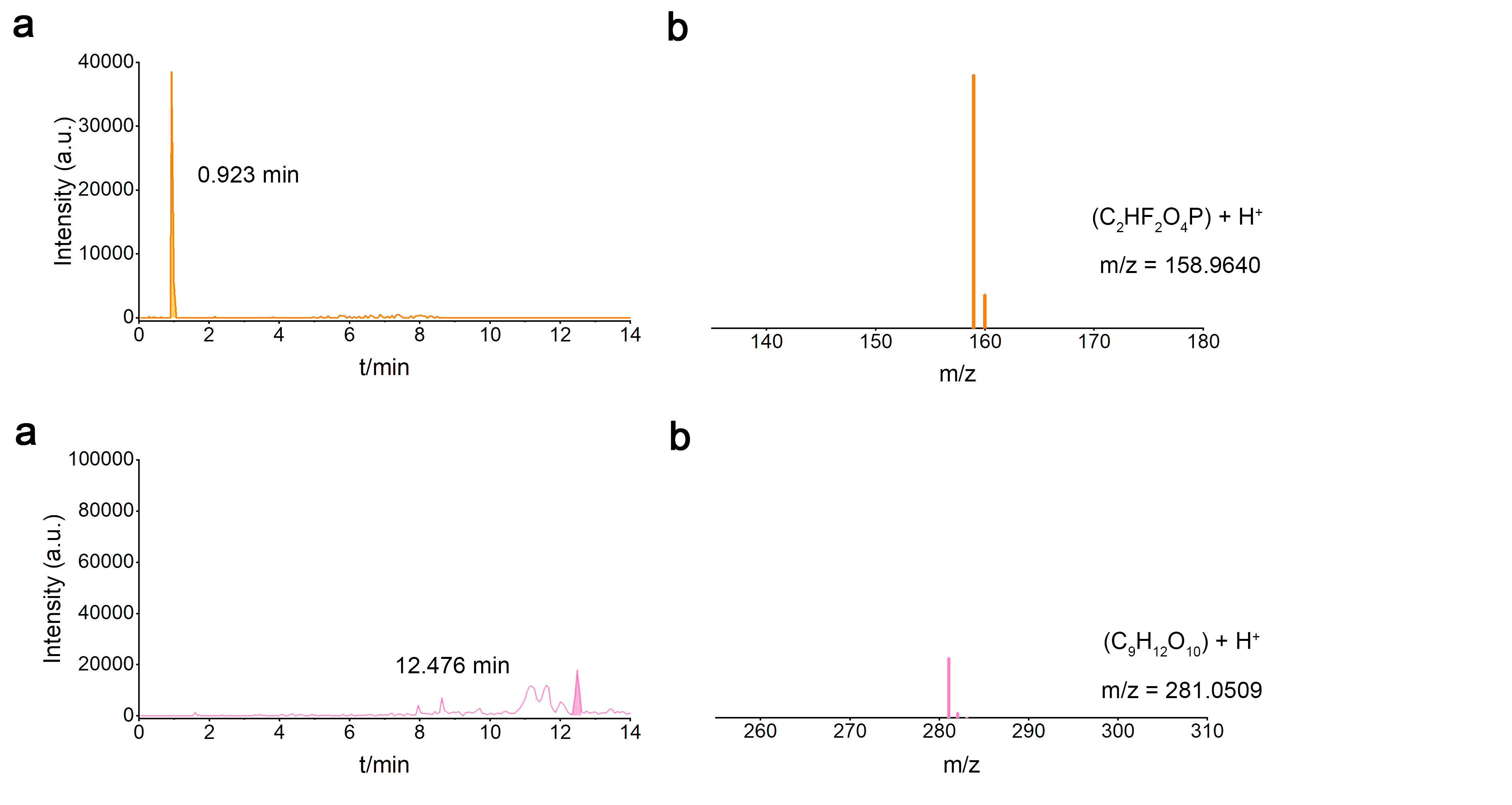


**Figure S11.** Identification of dissolution-resistant oxalate-containing species within CEI layer of NFM cathode. The related fragment ions and their corresponding mass-to-charge (*m/z*) ratios detected in the liquid chromatography-quadrupole time-of-flight mass spectrometry (LC-QTOF-MS) experiments. Liquid chromatography results in the leaching solution of the NFM cathode, with chromatographic retention times of 0.923 min. The mass-to-charge ratios of the corresponding molecular structures C₂HF₂O₄P have been annotated in the mass spectra. The distinguishing peaks within mass spectra are observed in positive ion mode. The limited solubility of oxalate-containing species contributes to the dissolution-resistant of the NaDFBOP-derived CEI layer.


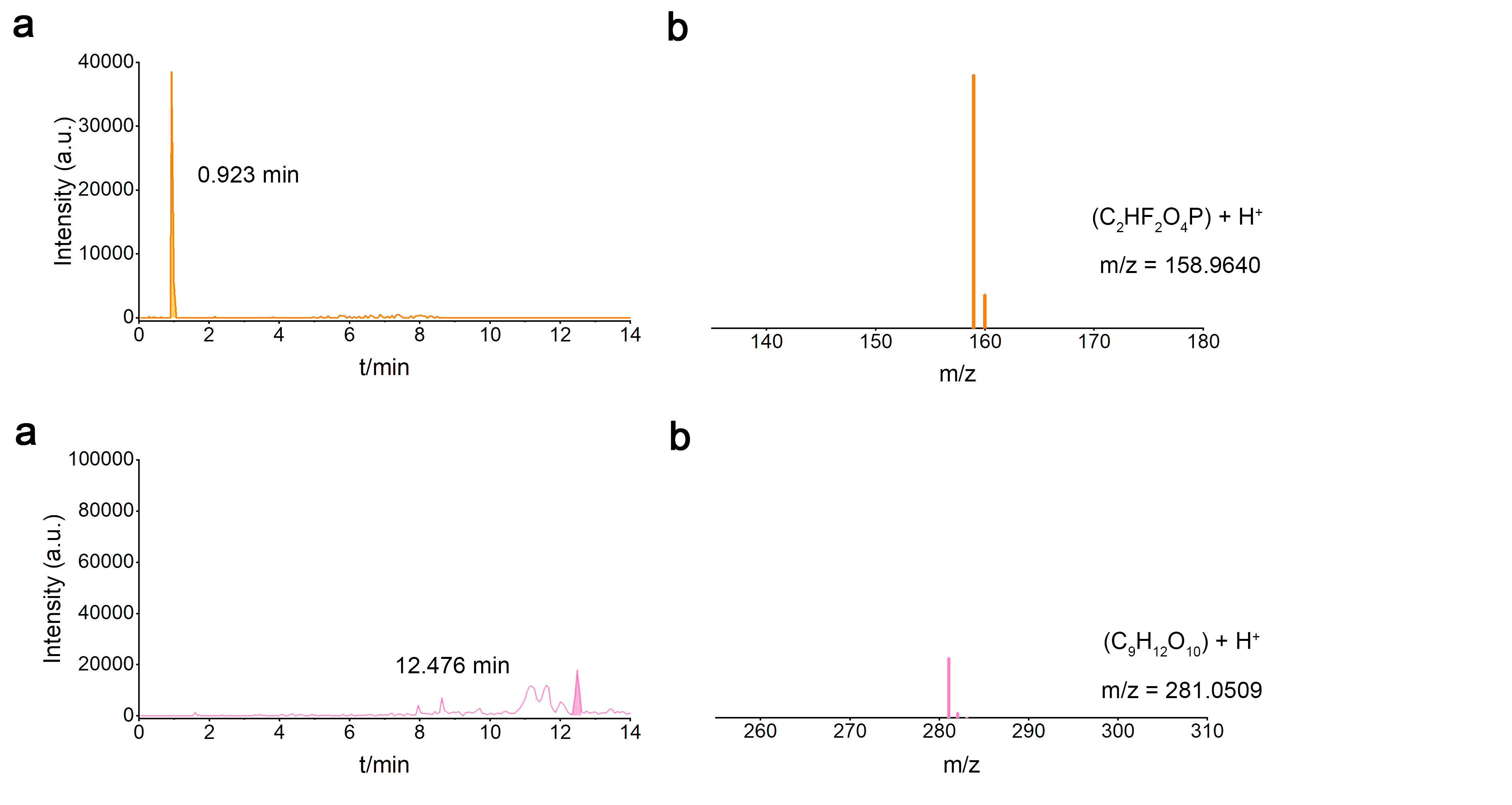


**Figure S12.** Identification of dissolution-resistant oxalate-containing species within CEI layer of NFM cathode. The related fragment ions and their corresponding mass-to-charge (*m/z*) ratios detected in the liquid chromatography-quadrupole time-of-flight mass spectrometry (LC-QTOF-MS) experiments. Liquid chromatography results in the leaching solution of the NFM cathode, with chromatographic retention times of 12.476 min. The mass-to-charge ratios of the corresponding molecular structures C₉H₁₂O₁₀ have been annotated in the mass spectra. The distinguishing peaks within mass spectra are observed in positive ion mode. The limited solubility of oxalate-containing species contributes to the dissolution-resistant of the NaDFBOP-derived CEI layer.


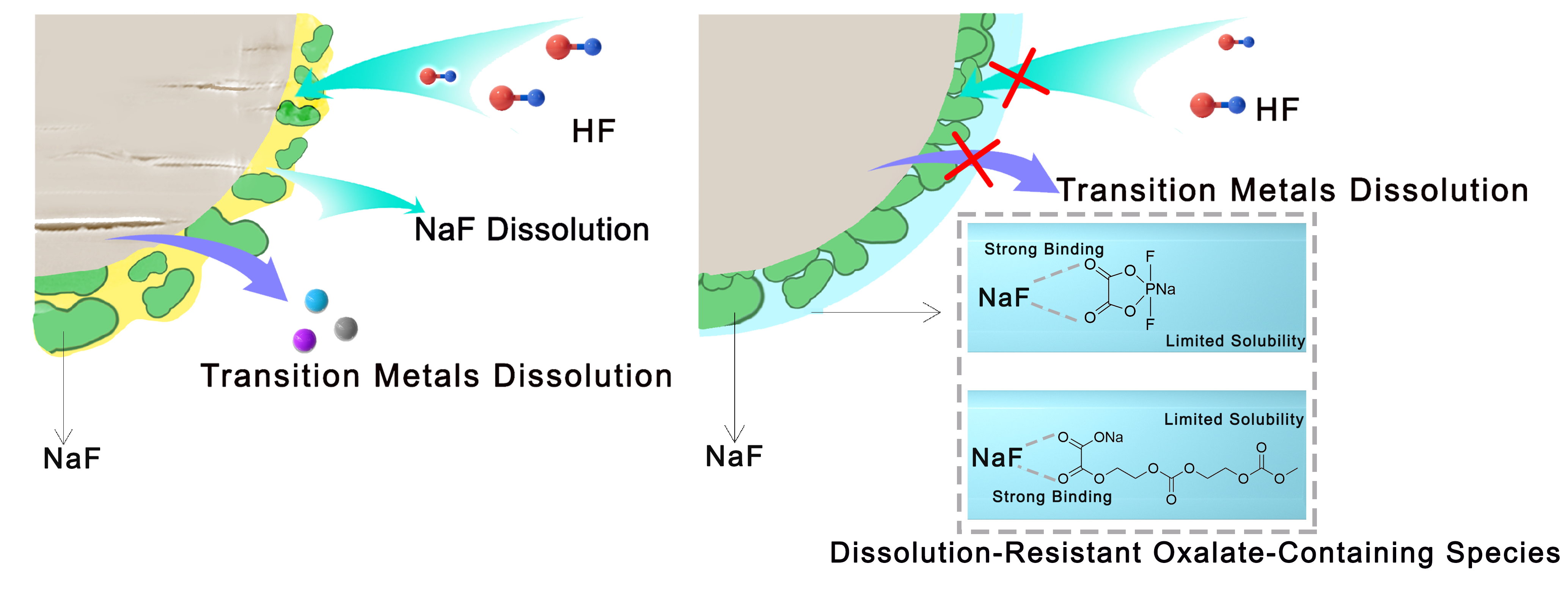


**Figure S13.** Illustration of the formation of a dissolution-resistant robust CEI layer on NFM cathode by the NaDFBOP additive.

The NaDFBOP additive also favors the formation of a robust CEI layer enriched of dissolution-resistant oxalate-containing species and inorganic NaF, which have strong mutual binding energy.

**Table S1.** Comparison of elevated temperature cycle life of NFM/HC full cells reported in literature.

| Cathode, anode | Electrolyte | Cycles, rate, voltage,and temperature | Capacity  retention | | Reference |
| --- | --- | --- | --- | --- | --- |
| **NaNi_1/3_Mn_1/3_Fe_1/3_O_2_**  **/HC full cells** | **1 M NaPF_6_ in EC/EMC (3:7)**  **1 wt. % NaDFBOP** | **500 cycles**  **1 C**  **1–4 V**  **50 °C** | **90.76%** | **This work** | |
| NaNi_1/3_Fe_1/3_Mn_1/3_O_2_  /HC full cells | 1 M NaPF_6_ + 0.5 M  NaFSI in EC, PC,  EMC and DEC with a  ratio of 1: 2: 6: 1  (by weight) | 80 cycles  1 C  2–4 V  50 °C | 82.30 % | 8 | |
| NaNi_0.5_Mn_0.5_O_2_  /HC full cells | 1 M NaClO_4_ in EC/EDC (1:1),  5 vol.% FEC | 100 cycles  0.5 C  1.9–4.2 V  55 °C  100 cycles  0.5 C  1.9–3.9 V  55 °C | 76.20 %  77.60 % | 9 | |

**Table S2.** Molecular dynamic (MD) simulations of NaPF_6_-EC-EMC, NaPF_6_-EC-EMC-NaDFBOP.

|  | NaPF_6_:EC:EMC=1:4.5:6.7 | | NaPF_6_:EC:EMC:NaDFBOP=1:4.4:6.7:0.06 |
| --- | --- | --- | --- |
| Number of NaPF_6_ per box | | 60 | 99 |
| Number of EC per box | | 270 | 440 |
| Number of EMC per box | | 402 | 660 |
| Number of NaDFBOP per box | | \ | 6 |
| Total number of atoms | | 9210 | 10122 |
| Simulation box size (Å^3^) | | 50.1×50.1×50.1 | 51.8×51.8×51.8 |
| MD, density (g/cm^3^) | | 1.186 | 1.192 |
| Bias temperature (K) | | 298 | 298 |

**References**

[1] Becke, A. D., Density‐Functional Thermochemistry. Iii. The Role of Exact Exchange, 1993, The Journal of Chemical Physics, 98 (7), 5648, https://doi.org/10.1063/1.464913

[2] Krishnan, R., Binkley, J. S., Seeger, R., Pople, J. A., Self‐Consistent Molecular Orbital Methods. Xx. A Basis Set for Correlated Wave Functions, 1980, The Journal of Chemical Physics, 72 (1), 650, https://doi.org/10.1063/1.438955

[3] Grimme, S., Ehrlich, S., & Goerigk, L., Effect of the damping function in dispersion corrected density functional theory, 2011, Journal of computational chemistry, 32(7), 1456–1465. https://doi.org/10.1002/jcc.21759

[4] Samoletov, A. A., Dettmann, C. P., Chaplain, M. A. J., Thermostats for “Slow” Configurational Modes, 2007, Journal of Statistical Physics, 128 (6), 1321, https://doi.org/10.1007/s10955-007-9365-2

[5] Berendsen, H. J. C., Postma, J. P. M., van Gunsteren, W. F., DiNola, A., Haak, J. R., Molecular Dynamics with Coupling to an External Bath, 1984, The Journal of Chemical Physics, 81 (8), 3684, https://doi.org/10.1063/1.448118

[6] P. P. Ewald, Ann. Phys. 1921, *369*, 253-287.

[7] M. P. Tosi, Solid State Phys. 1964, *16*, 1-120.

[8] Fan, W., Wang, W., Xie, Q., He, X., Li, H., Zhao, J., et al., A Sodium Bis(fluorosulfonyl)Imide (NaFSI)-based Multifunctional Electrolyte Stabilizes the Performance of NaNi_1/3_Fe_1/3_Mn_1/3_O_2_/Hard Carbon Sodium-Ion Batteries, 2024, 30 (43), e202401321, https://doi.org/https://doi.org/10.1002/chem.202401321

[9] Wu, Y.-B., Hu, H.-Y., Li, J.-Y., Dong, H.-H., Zhu, Y.-F., Chen, S.-Q., et al., Periodic Law-Guided Design of Highly Stable O3-Type Layered Oxide Cathodes for Practical Sodium-Ion Batteries, 2025, Chemical Science, 16 (9), 3928, https://doi.org/10.1039/D4SC08351B
